# Genome degradation in plant tissue culture

**DOI:** 10.1073/pnas.2530182123

**Published:** 2026-04-22

**Authors:** Matthew W. Davis, Charles A. Leslie, Chaehee Lee, Evan Long, Li Meinhold, Megan Lorenc, Franklin Lewis, Patrick J. Brown, Grey Monroe

**Affiliations:** ^a^Department of Plant Sciences, University of California, Davis, CA 95616; ^b^Department of Agriculture–Agricultural Research Service, Northwest Irrigation and Soils Research Laboratory, Kimberly, ID 83341

**Keywords:** somatic mutation, tissue culture, genome instability, tissue-layer specificity, somatic recombination

## Abstract

Many plants, unlike most animals, can reproduce as clones [D. Kester, *HortScience* **18**, 831–837 (1983), 10.21273/hortsci.18.6.831]. Cloning plants is vital to agriculture and biotechnology [H. Barton, T. Denham, *Quatern. Int.* **489**, 17–25 (2018), O. L. Gamborg, *In Vitro Cell. Dev. Biol. Plant* **38**, 84–92 (2002), K.-H. Neumann, A. Kumar, J. Imani, *Plant Cell and Tissue Culture—A Tool in Biotechnology: Basics and Application* (ed. 2, 2020)], but the extent to which somatic mutation arises during asexual propagation remains an outstanding question. Leveraging a natural experiment of walnut clones spanning five decades and multiple cloning methods, we found a complex history of somatic mutation revealing developmental cell dynamics. A haplotype-phased reference genome exposed extreme genomic instability, a distinct mutation spectrum, and a >3,500% increase in mutation rate within clonal somatic embryos. Pairing these mutations with the haplotype-phased assembly revealed the canonical flowering plant meristematic layers from bulk tissue sequencing and uncovered frequent fixation events throughout development. These findings raise concerns about genomic integrity in tissue culture, test assumptions about mutagenesis in clonal propagation, and establish a benchmark for emerging paradigms in somatic mutation research.

## Significance

Plants can be clonally propagated in a variety of ways, and plant clones have been used in agriculture for millennia. The in vitro cloning of plants is a critical component of plant biotechnology, enabling transformation and gene-editing. Though plant cloning is vital to biotechnology and agriculture, it is uncertain the impact mutation has in different clonal propagation techniques. We uncover somatic embryo clones have a >3,500% increase in mutation rates and exhibit whole chromosome duplications, large-scale deletions, somatic recombination events, and increased transposable element activity. Additionally, we use somatic mutation to reveal development, showing somatic embryos undergo frequent fixation and detecting tissue-layer specific mutation. This work raises concerns about the genetic integrity of plants used for biotechnology approaches.

Unlike most animals, many plants can be readily propagated as genetic clones (1). Cloning has been a part of agricultural practices for thousands of years (2, 3), and millions of plants are now clonally propagated annually. This asexual propagation facilitates the large-scale proliferation of preferred genotypes, but the extent to which genetic integrity is maintained in the face of somatic mutation remains an open question. Clonal propagation is also critical for biotechnology, as it is an essential intermediate step in producing transgenic and gene-edited plants for many species (4–8). Investigating somatic mutation in clonally propagated plants at whole-genome scales is critical for answering long-standing questions of fundamental and economic importance.

Plants can be cloned through various in-field and in vitro methods, including budwood cuttings, shoot culturing, and somatic embryogenesis (9). Despite being clonally generated and assumed a priori to be genetically identical, plant clones occasionally display new and variable phenotypes, known as somaclonal variation (10, 11). This can result in poor field performance (12–14), but it also may give rise to new crop varieties, as seen in wine grapes and oranges (15). While some somaclonal variation is due to epigenetic differences (16) or desilencing of transposons (17), much of it is likely due to de novo point mutations (18) and other genomic changes. Here, we leverage genomic sequencing technologies in walnut clones generated and maintained with different clonal propagation techniques to compare somatic mutation rates across time and space.

Somatic mutation in multicellular organisms creates natural mosaics of genetically distinct cell populations (19). In mammals, somatic mosaicism has been investigated extensively to study cell lineage dynamics and development (20), while similar investigations in plants represent a burgeoning frontier (21–23). This mosaicism can present a significant challenge in plant biotechnology, as plants regenerated after transformation and gene editing are frequently chimeric (24). To mitigate this, somatic embryos are often used, as they are less prone to chimerism than organs derived through organogenesis (25). Substantial effort has been focused on improving transformation and editing efficiencies, yet the general genomic integrity of the clonal material warrants further attention. Incorporating somatic mosaicism as a framework for studying mutation in plants offers a powerful lens for uncovering fundamental plant biology and advancing methods in plant biotechnology.

To address these critical knowledge gaps, we combined short- and long-read sequencing to measure somatic mutation in a unique clonal walnut (*Juglans regia*) lineage established in the 1970s. These data show extremely elevated mutation and genomic instability in plant tissue culture, while simultaneously revealing clear developmental insights. This study represents the most exhaustive exploration of somatic mutation in plants, capturing mutations from the single nucleotide level to whole-chromosome scales, leveraging mutational mosaicism to reveal developmental biology, and providing a benchmark for assessing mutation in plants.

## Results and Discussion

### Mutation Accumulation across Clones.

Since the 1970s UC Davis has maintained clones of the world’s most popular walnut cultivar, “Chandler.” In 1995, a single clonal somatic embryo was generated from Chandler anther tapetum and multiplied in culture by repetitive direct somatic embryogenesis to serve as a biotechnology resource (26). Over the next three decades this culture underwent expansions and contractions, existing now as 12 subpopulations, all clonally derived from the single somatic embryo progenitor. This population provided us with a unique opportunity to investigate long-term somatic mutation.

**Fig. 1.**
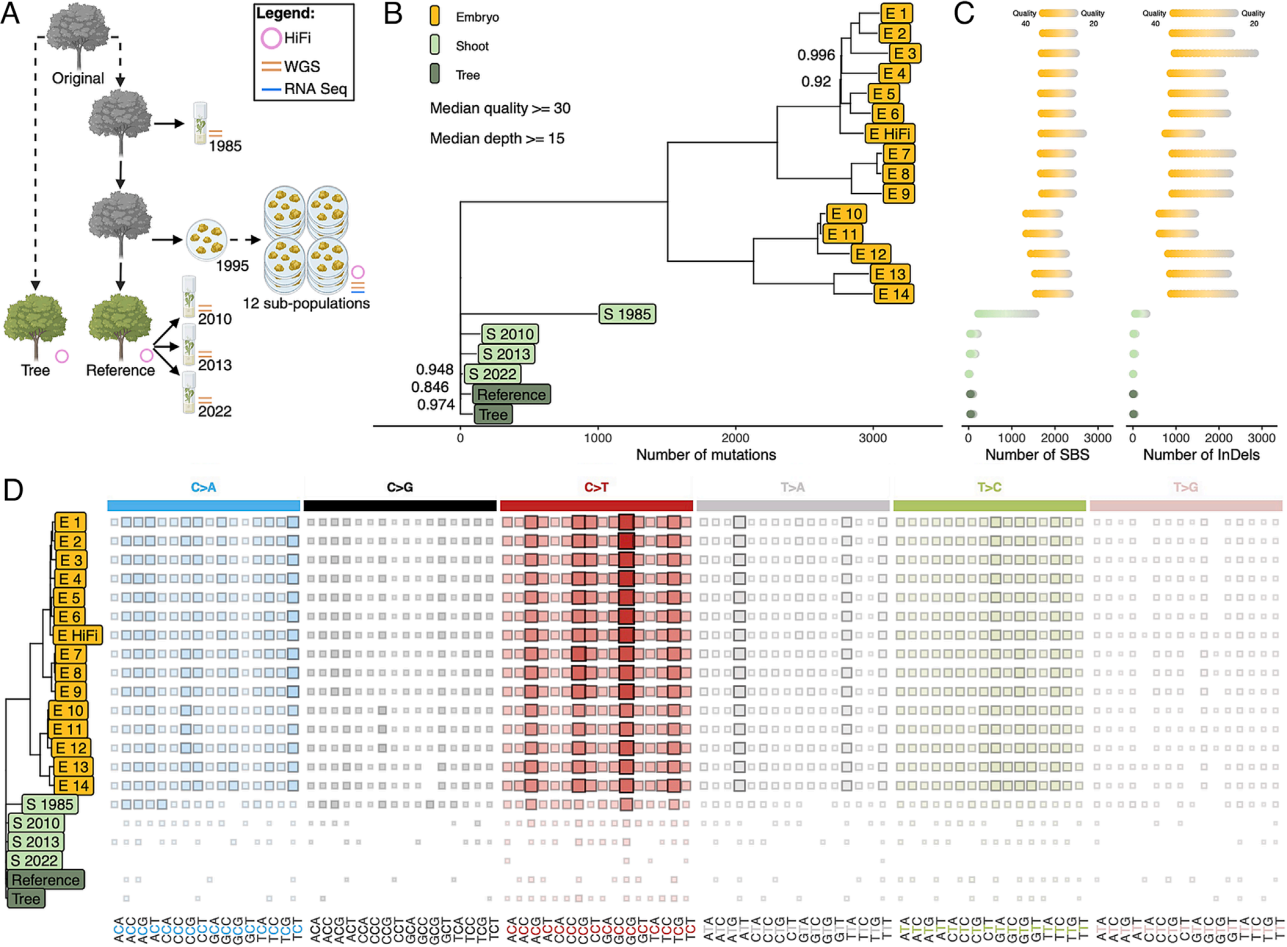
Somatic embryos exhibit high levels of mutation compared to shoots and trees. (*A*) Schematic of the relationship among all clones. Icons denote trees, shoot cultures, and somatic embryos. Gray icons represent dead clones. Dashed lines indicate an unknown number of clonal propagations, solid lines indicate a single clonal propagation. (*B*) Phylogeny of all sequenced clones constructed from de novo somatic single base substitutions (SBS) and InDels. Branch length is proportional to the number of mutations. Bootstrap support less than 1 is displayed. (*C*) The number of SBS and InDels present in each sample at minimum median quality scores of 20 to 40, in increasing intervals of 1. (*D*) The mutation spectra of SBS in the clones. Larger squares and darker color indicate higher occurrence of each context.

Our initial goals were twofold: to conduct a benchmark study of somatic mutation while addressing the long-standing question of how mutation impacts clonal plants. We began in 2021 by sequencing two field-grown trees with PacBio HiFi and two somatic embryos from the same subpopulation with Illumina Whole Genome Sequencing (WGS) (Fig. 1*A* and Dataset S1).

Combining HiFi and chromatin-capture Omni-C data, we constructed a telomere-to-telomere haplotype-phased genome assembly for the walnut cultivar Chandler from the same tree used to construct the previous reference genome assemblies (27, 28). The phasing accuracy of the assembly was confirmed using a genetic map (*SI Appendix*, Figs. S1–S6 and Datasets S2–S4). All in vitro clonal material was either introduced from this reference tree or its direct clonal progenitors (Fig. 1*A*) and are considered the same age, as they were derived from the same zygotic event.

Using a strict filtering strategy to remove ancestral heterozygosity, sequencing errors, and mapping artifacts, we identified a set of high confidence de novo mutations (*Materials and Methods*, *SI Appendix*, Fig. S7 and Dataset S5). The two somatic embryos exhibited a surprising >3,500% increase in mutation when compared to the trees (median quality ≧30, median depth ≧15). To confirm this unexpected result, we performed PacBio HiFi sequencing on a random somatic embryo from a random subpopulation, as well as Illumina WGS and RNA sequencing on a random somatic embryo from each of the 12 subpopulations. We also used Illumina WGS on clones generated with a different in vitro approach, sequencing shoot cultures introduced from nodal cuttings in 1985, 2010, 2013, and 2022 (Fig. 1*A* and Dataset S2). Mutations in these samples were subsequently identified with the same pipeline.

We created a phylogeny of de novo mutations in all clones (Fig. 1*B*), which further confirmed the >3,500% somatic mutation enrichment across embryo samples relative to the reference tree. The somatic embryos formed distinct clades, indicating the shared ancestry of the subpopulations. The enrichment of somatic mutations in the embryos remained true regardless of the sequencing method (i.e., short- vs. long-read) or variant filtering parameters (Fig. 1 *B* and *C*) and provides insight into the propagation history of the somatic embryos since their introduction three decades ago.

The mutations in the somatic embryos reflected similar functional classes to the shoot cultures and trees (*SI Appendix*, Fig. S8 and Dataset S6), and their elevated mutation rate could not be explained by relaxed purifying selection, despite fewer mutations observed within gene bodies compared to intergenic regions. This is consistent with patterns of neutral somatic mutation in other plants and greater DNA repair efficiency in gene bodies (29, 30) (*SI Appendix*, Fig. S9*A*). Time and cell division rate are both important factors in somatic mutation accumulation (31–36). While we cannot separate the effect of replication-dependent mutation from replication-independent mutation over time, we use annual time as a comparative metric, effectively combining them in the rate denominator. When compared to annualized somatic and germline mutation rates in other plant species, the mutation rate in the somatic embryos was higher, though similar to the rate detected in rice callus, another tissue culture cloning method (37–40) (*SI Appendix*, Fig. S9*B*). The elevated mutation rate per unit time in the embryos may result from either a higher number of mutations per cell division, an equivalent number of mutations per cell division accompanied by an increased cell division rate, or a higher number of mutations per cell division and a higher cell division rate in the embryos relative to the trees and shoots.

In the shoots, the number of mutations scaled linearly with time in culture (*SI Appendix*, Fig. S9*C* and Dataset S5), with the longest maintained shoot culture (S 1985) showing a >1,000% increase in mutation compared to the trees (median quality ≧30, median depth ≧15). This pattern of somatic mutation accumulation increasing with time is comparable to what has been observed in mammalian somatic tissues (32, 33), though the tree clones sequenced had lower yearly single base substitutions (SBS) and InDel accumulation rate estimates than mammals (33) (*SI Appendix*, Fig. S9 *D* and *E*).

The extreme differences in mutation accumulation were also reflected in the mutational spectra, as all somatic embryos exhibited a similar profile that was distinct from that of S 1985 (Fig. 1*D* and *SI Appendix*, Fig. S10), despite somatic embryogenesis and shoot culture both being in vitro methods. The remaining shoot cultures and trees had too few mutations to individually assess differences in spectra (Fig. 1*D* and Dataset S5). We calculated the similarity of each spectra to known mutagenic SBS profiles (41) and found some differences in the top matching profiles in the embryos compared to the shoots and trees (Dataset S7).

Though these clonal samples all originate from the same zygote and are the same age, they exhibit vast differences in mutation rate. These results shed light on the mutagenic potential of tissue culture, with the mutation burden depending on cloning technique and time maintained in culture. The spectrum of mutation also appears to be dictated by the clonal propagation method, implying variability in DNA damage and repair, while suggesting developmental differences between the clones.

### Large-Scale Genomic Instability.

Somatic chromosomal instability has been observed in cancer (42), human somatic cells (43), and various plant tissues (44–47), and has the potential to impact the fidelity of clonal propagation in agriculture and biotechnology.

**Fig. 2.**
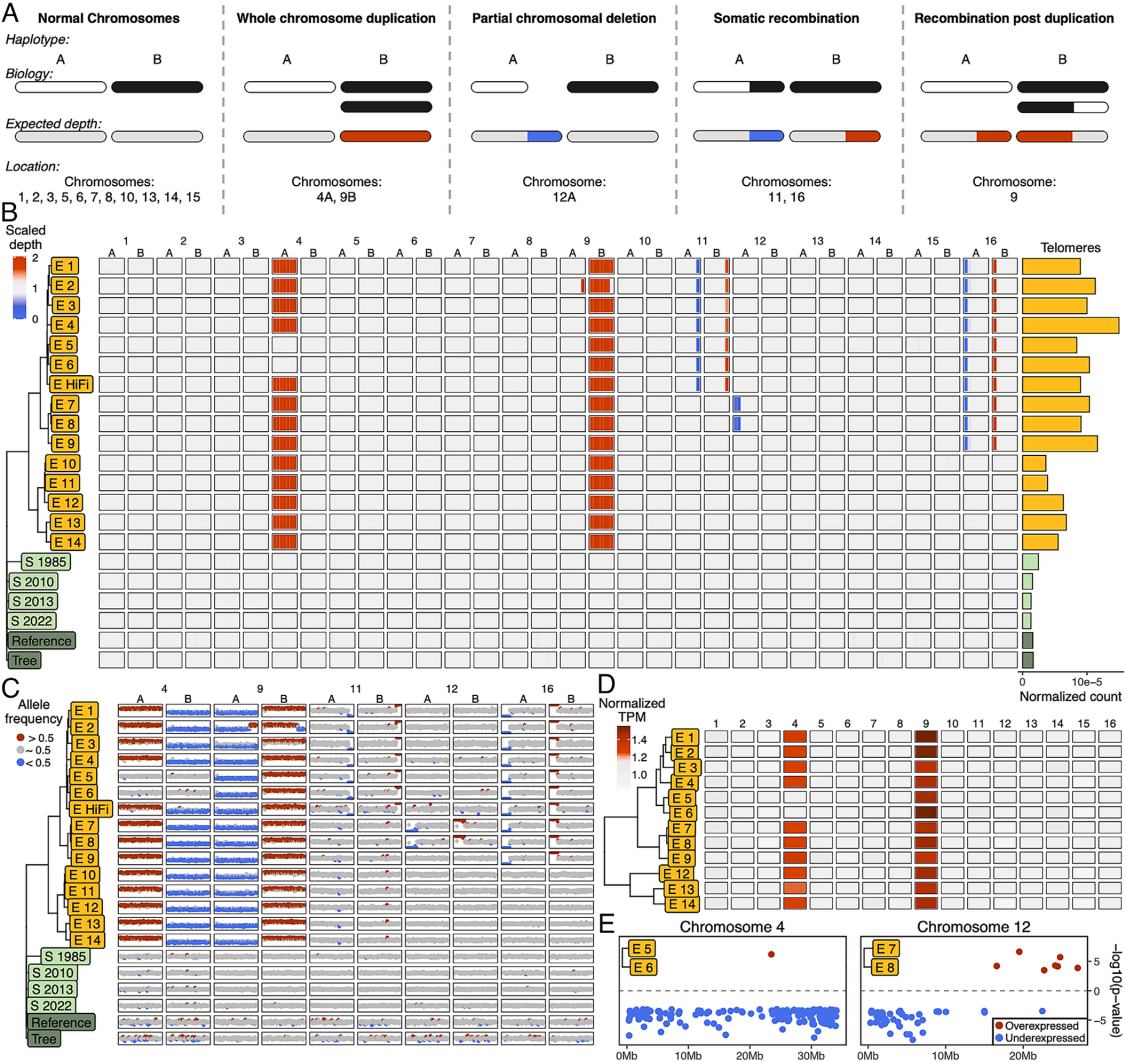
Genomic instability in the somatic embryo clones. (*A*) Read depth schematic. (*B*) Scaled read depth of ancestral heterozygosity in every chromosome of every clone. Red and blue represent high and low sequencing depth, respectively. Bars to the right display the number of telomeric repeats corrected by sequencing depth. (*C*) Allele frequency of ancestral heterozygosity in chromosomes exhibiting genomic instability. Red and blue represent windows with mean allele frequency significantly greater or less than 0.5, respectively. (*D*) Normalized expression of entire chromosomes in the somatic embryos. Red indicates higher than average expression. (*E*) Comparison of individual gene expression across clades of embryos. Red and blue points are comparatively over- and underexpressed genes, respectively.

We assessed genomic instability with the sequencing depth of phased ancestral heterozygosity across clonal samples (Fig. 2*A* and *SI Appendix*, Fig. S11*A*). All somatic embryos carry a duplication of chromosome 9 haplotype B, and all but two carry a duplication of chromosome 4 haplotype A (Fig. 2*B*). We verified the most parsimonious explanation of the absent trisomy using allele frequency: The 4A duplication occurred once and was lost in the E 5 & E 6 clade, evidenced by the retention of 4A specific mutations that occurred after the chromosome duplication in these individuals (*SI Appendix*, Fig. S11 *B–D* and Dataset S8). We dated the 4A and 9B trisomies using shared de novo mutations and found ~10-fold fewer mutations occurred before the duplication on 9B compared to 4A, indicating 9B duplicated much earlier in the somatic embryo’s history (*SI Appendix*, Fig. S11*E* and Dataset S9). Additionally, there are incredibly few shared mutations on the duplicated 9B haplotype, suggesting that the duplication occurred either in the source material before the induction of embryogenesis or shortly after. Since the generation of this somatic embryo in 1995 (26), the walnut improvement program has been unsuccessful in initiating new Chandler somatic embryo cultures despite numerous efforts, and the early onset of this duplication hints that it may be important for successful somatic embryogenesis in this genotype.

A partial chromosomal deletion of 12A, a somatic recombination between the chromosome 9 haplotypes in the trisomy, a somatic recombination between the chromosome 11 haplotypes, and a somatic recombination between the chromosome 16 haplotypes were also found in the somatic embryos (Fig. 2*B* and *SI Appendix*, Fig. S12*A*). Notably, there was no large-scale structural variation in the shoot cultures and trees.

We verified the instabilities using allele frequency and sequencing depth (Fig. 2*C* and *SI Appendix*, Fig. S12*B*) and further explored their effects on gene expression in the somatic embryos. The duplicated chromosomes exhibited higher gene expression relative to diploid chromosomes (Fig. 2*D*), and the clades of somatic embryos lacking the 4A duplication or with the 12A deletion exhibit lower expression. Considering these structural variants (SVs) alone, we identified thousands of genes whose expression is affected by somatic mutation accumulation.

Telomeric repeats were identified in every sample (*SI Appendix, Materials and Methods*), and the somatic embryos had more telomeric repeats than the shoot and tree clones (Fig. 2*B* and *SI Appendix*, Fig. S13*A* and Dataset S10). Interestingly, one of the two major clades of somatic embryos had more telomeric repeats than the other clade, and genes associated with telomere maintenance were more highly expressed in this clade (*SI Appendix*, Fig. S13 *B*–*E*). S 1985 also had more telomeric repeats when compared to the other shoot cultures and trees, and telomere length in the shoots appears to correspond with time in culture (*SI Appendix*, Fig. S13*F*).

All instances of genomic instability in the somatic embryos are congruent with the SBS and InDel phylogeny, giving us additional confidence in our results and further insight into the clones’ shared history. These analyses also provide additional evidence that recombination occurs in plant somatic cells, a phenomenon that has been proposed to occur through the life cycle of a plant (48, 49), as a DNA repair response (48, 50), or as a pathogen stress response (51). The absence of instability in the shoot cultures and trees further underscores the mutational consequences of specific clonal propagation methods.

Clone age has been associated with reproductive success (52), and these genomic instabilities impact the expression of thousands of genes. After several attempts, we managed to germinate a few randomly selected somatic embryos in July 2024, but they display a different developmental morphology than traditional shoot cultures (*SI Appendix*, Fig. S14), potentially due to the severity of the mutation accumulation and genome instability.

### Transposable Element (TE) Expansion.

Barbara McClintock speculated on the genomic stressors associated with plant tissue culture (53), and TE activation in these conditions is well documented (17, 54, 55). Yet until the advent of long-read sequencing, the ability to comprehensively analyze genome-wide TE expansion was limited. Leveraging PacBio HiFi and our haplotype-resolved assembly, we investigated de novo somatic TE expansion in the clones.

**Fig. 3.**
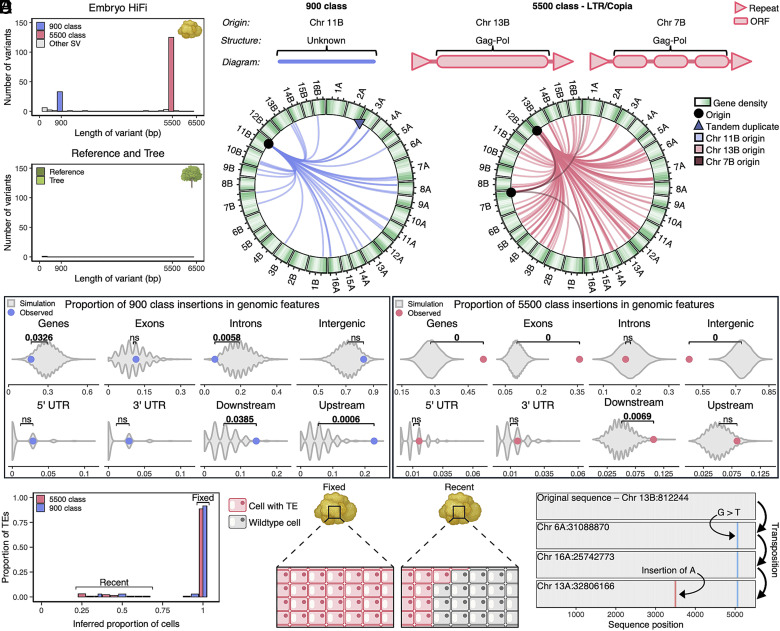
TEs are continuously active in the long-read somatic embryo. (*A*) Frequencies of de novo insertions in the clones sequenced with PacBio HiFi. (*B*) Morphology of class 900 and 5,500 transposons. (*C*) Circos plots depicting transposition of 900 and 5,500 class TEs. Lines trace TE movement in the somatic embryo from the putative origin to de novo insertion sites. (*D*) A comparison of the observed location of TE insertions relative to genomic features. Purple points indicate the observed means of the 900 class TEs and pink points indicate the observed means of the 5,500 class TEs. Gray violins represent the distribution of means of 10,000 random simulations. Brackets are labeled with statistically significant differences (*P* < 0.05) between observed and simulated means. (*E*) Class 900 and 5,500 TEs observed in different proportions of cells inferred from allele frequency that compose the somatic embryo, accompanied by a diagram. (*F*) Tracking a class 5,500 TE through multiple transposition events using mutation accumulation.

Analysis of de novo SVs in the long-read samples revealed enrichments of large insertions in the E HiFi somatic embryo around 900 bp and 5,500 bp in length, along with a lack of insertions in the trees (Fig. 3*A*). These high frequency insertions were termed 900 class and 5,500 class respectively. The 5,500 class was composed of two main sequences, both identified as Copia/LTR transposons, while the 900 class was composed of one sequence with no readily identifiable domains (Fig. 3*B* and *SI Appendix*, Figs. S15 and S16). In both classes, insertions were accompanied by target site duplications of ~10 bp (*SI Appendix*, Figs. S17 and S18).

The putative origins of these insertions were identified based on sequence identity in the haplotype-phased reference assembly, and the transpositions in the somatic embryo were tracked across the genome. There were 35 new 900 class insertions and 131 new 5,500 class insertions (Fig. 3*C*). We found significant bias in insertion sites for both elements, with 900 class insertions enriched in regions up- and downstream of gene bodies and underrepresented within genes and introns (Fig. 3*D*). The 5,500 class insertions showed an enrichment within genes, exons, and downstream of gene bodies, but were underrepresented in intergenic space (Fig. 3*D*). The insertion bias patterns of the 5,500 class are consistent with known patterns of insertion exhibited by Copia/LTR (56) retrotransposons. We did not find these same biases for non-TE-like insertions (*SI Appendix*, Fig. S19 *A*–*C*). Purifying selection would be expected to remove genic TE insertions, therefore it is unlikely to explain the observed patterns of TE bias. Conversely, SBS and InDels are reduced in genic sequences, consistent with previous findings on somatic mutation in both plants and humans (29, 30, 32, 57) (*SI Appendix*, Fig. S9*A*).

To assess whether TEs are continuously active in the somatic embryo, TE insertions were called against the haplotype-phased assembly, allowing us to directly infer the proportion of mutated cells. While most TE insertions are present in all cells (100% allele frequency), we detected phased sites with insertions that exist within only a fraction of cells, a result consistent with ongoing TE activity (Fig. 3*E* and *SI Appendix*, Fig. S19*D*). We also observed sequential transposition through the accumulation of novel mutation, indicating a TE insertion, followed by a mutation event, then subsequent insertion (Fig. 3*F* and *SI Appendix*, Fig. S20 and S21). In the embryos with short-read sequencing, we identified hundreds of 5,500 class insertions genome-wide, which did not exist in the shoot cultures and tree clones (*SI Appendix*, Fig. S22). Both the 900 class and 5,500 class TEs were expressed in every somatic embryo (Dataset S11).

Our results show that various TEs are continuously active in the somatic embryos. The 900 class insertions exhibit a lack of identifiable motifs, but retain signatures of target site duplication. This suggests they are nonautonomous, though we found no evidence of the corresponding autonomous element and the 5,500 class does not share sequence similarity with the 900 class.

Increased transposon activation may reduce the efficacy of using somatic embryos in plant biotechnology, as continual TE mutagenesis may break genes or alter their expression (58).

### Somatic Mutation Reveals Development.

Previous work in plants has leveraged somatic mutation to reveal developmental dynamics (59), and next generation sequencing has clarified questions about human ontogenesis (20, 60). Using somatic mutation, we explored the developmental underpinnings of clone variability.

The meristems of flowering plants are organized into three distinct cell layers termed L1, L2, and L3 (61–63), with mutations typically restricted to individual layers (21, 23). However, the boundary between L2 and L3 is notably less stable than the boundary between L1 and L2 (63–69). Importantly, allele frequency impacts the detectability of mutations. Lower frequency mutations are more likely to be undetected, and because the L1 tissue layer is composed of fewer cells it is more challenging to detect mutations constrained to this layer in bulk sequencing data. Somatic embryogenesis in plants may occur by polyclonal founding followed by iterative fixation events (36), or via a single founder cell (70–72). Both developmental options would lead to mutations detected in all cells of the newly generated somatic embryo. In somatic embryo studies of *J. regia* and *Arabidopsis*, newly generated embryos have been shown to originate from a single epidermal cell (72–74).

**Fig. 4.**
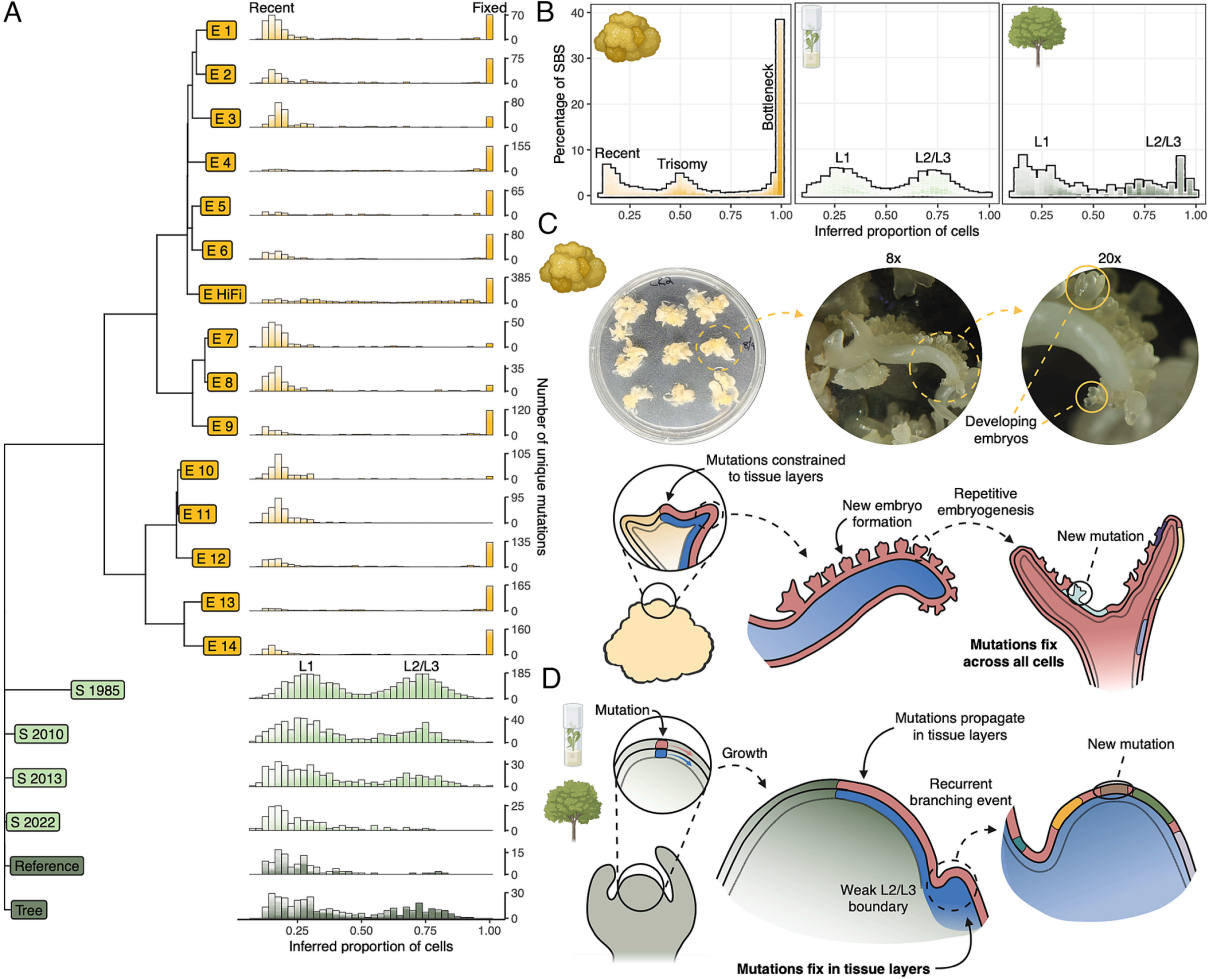
Development and anatomy contribute to differences in mutation burden between clones. (*A*) Number of singleton de novo SBS and InDels occurring in a proportion of the cells inferred from allele frequency composing each clone. Plots were generated from minimum quality scores of 5 to 40 and plotted on top of one another with alpha = 0.1. Confidence in the mutation count is therefore depicted by bar darkness, with darker and lighter regions representing higher and lower confidence, respectively. (*B*) The percentage of de novo SBS within each bin, grouped by the clonal propagation method. Confidence in mutation percentage is depicted by darkness of the bar. (*C*) Diagram and microscopy depicting somatic embryo growth and development. Somatic embryos at different developmental stages are labeled in the microscope image and a proposed mechanism of somatic mutation fixation in the somatic embryos is presented in the diagram. (*D*) Schematic portraying a proposed mechanism of mutation fixation within tissue layers in the shoot culture and field-grown tree clones.

Mutations were called against the phased assembly and restricted to de novo SBS and InDels unique to individual clones. As with the TE analysis, this allowed us to directly infer the fraction of cells carrying de novo mutations. The frequency of these mutations revealed two distinct patterns. Most mutations in the somatic embryos are present in all cells, while some mutations are present at low frequency (Fig. 4 *A* and *B*). The unique fixed mutations at 100% allele frequency reveal that all cells trace to a single clonal cell lineage, and the high number of these mutations indicate fixation events are common in the somatic embryos. This fixation likely occurs when the somatic embryos bud from the L1 layer (72, 73), and the low frequency mutations that are not present in all cells suggest this process occurs often (Fig. 4 *B* and *C* and *SI Appendix*, Fig. S23*A*).

Conversely, we found essentially no mutations fixed in all cells within the shoot cultures and tree clones, which instead exhibit a distinct bimodal distribution of somatic mutation frequencies (Fig. 4 *A* and *B*). This pattern would be expected from bulk sequencing of two developmentally distinct sources, such as angiosperm tissue layers, and fixation within those layers (36). The impermanence of the boundary between the L2 and L3 layers allows for the propagation of a mutation in both layers, which constitute a higher fraction of cells in the organism than mutations constrained to L1 (Fig. 4 *B* and *D* and *SI Appendix*, Fig. S23*B*). The L1 layer gives rise to the leaf epidermis, while the L2 layer gives rise to the internal tissue of the leaf, leading to more cells derived from L2 in bulk sequencing data (75).

Somatic mutation in long-term clones provides a valuable resource to study anatomy and development in plants. Mutations fixed in all embryo cells reveal that the embryos derive from a single clonal cell lineage and that these fixation events happen frequently. Simultaneously, the mutations at low frequency shed light on the ongoing nature of this process. We were also able to rediscover long-studied anatomy of flowering plants by tracking somatic mutations across a haplotype-phased genome assembly. These developmental differences are likely important factors underlying the mutation variation among clonal propagation methods.

### Conclusion.

Given the widespread use of cloning in nature, agriculture, and biotechnology, plants represent one of the most critical systems for understanding the dynamics of somatic mutation and its consequences.

Tissue culture is an essential part of genome engineering in many plants, where methodological advancements have largely prioritized increased transformation and editing efficiencies. Our findings suggest another crucial area of optimization: minimizing mutation accumulation to preserve genomic integrity. Somatic embryos are often used for genome editing because they avoid chimerism, likely due to the frequent fixation of single clonal cell lineages we revealed. However, we also found somatic embryos exhibit a high mutation rate and extreme genomic instability. The focus on editing efficiency may unintentionally select for systems with higher mutation accumulation.

These somatic embryos have been maintained in culture for several decades, and there is evidence of continual mutation accumulation over time. We predict that the extreme mutation burden we observed could be ameliorated in material subject to shorter in vitro periods, however we also find potential evidence of whole-chromosome duplication occurring during the initial stages of culture. Sequencing regenerated material to facilitate the early detection of potentially deleterious mutations is advised to avoid advancing and propagating suboptimal clones. While somatic mutations may impact the viability of the plants, they are exceedingly unlikely to have adverse effects if consumed.

In addition to these practical implications, our observations address fundamental questions about mutation in plants. We identified distinct mutational differences among clonal material, from single base changes to whole-chromosome duplications. These findings inspire future work to investigate how differences in DNA damage, repair, and development drive somatic mutation dynamics through time and space in plants.

## Materials and Methods

### Calling SBS and Small InDels.

Short read quality was assessed using FastQC v0.11.9 (76) and reads were trimmed using Trimmomatic v0.39 (77). Trimmed reads were then aligned to the primary reference assembly and the concatenated haplotype assembly using Minimap2 v2.17-r941. The resulting binary alignment map (BAM) files were sorted, duplicates were marked, and the files were indexed with SAMtools v1.13 (78). SBS and InDels were called against the primary and concatenated genome assemblies using DeepVariant v1.6.1 (79) with the model type “WGS” to generate variant call format (VCF) and genomic variant call format (GVCF) files. For regions within the GVCF that contained no variants, median depth of the region was reported.

Long reads were aligned to the primary assembly and the concatenated haplotype reference assembly using the PacBio Minimap2 wrapper pbmm2 v1.13.1 (https://github.com/PacificBiosciences/pbmm2) with the preset “HIFI.” The resulting BAM files were sorted and indexed using SAMtools v1.13. SBS and InDels were called against the primary and concatenated assemblies using DeepVariant v1.6.1 with the model type “PACBIO” to generate VCF and GVCF files. For regions within the GVCF that contained no variants, median depth of the region was reported.

The VCFs were additionally annotated in R with custom scripts. Sites were assessed for mapping probability. Reference allele and alternate allele depths were extracted, and the total depth of a site was recalculated by adding the reference allele depth and the alternate allele depth. The variant allele frequency of a site was recalculated by dividing the alternate allele depth by the recalculated total depth. Median depth was calculated across individuals at the same position, as was median quality.

### Identifying de Novo Mutations.

To identify small-scale de novo mutation and ancestral heterozygosity, all primary assembly sample VCFs were organized into groups based on tissue type and propagation method. All somatic embryos were classified as “embryo” group, all shoot cultures were classified as “shoot” group, and all field-grown trees were classified as “tree” group. Variants that were found in more than one group were considered to be ancestral heterozygosity. Variants found only within the embryo group were determined to be de novo mutations, as all embryos are derived from one initial embryo, and removing mutations shared by the embryos would be removing real de novo mutations. Variants found between members of the same group in the shoot and tree groups were removed, as these propagations were independent of one another and shared mutation was likely ancestral heterozygosity. The remaining mutations in these individuals were determined de novo (*SI Appendix*, Fig. S7). A similar assessment of the combined haplotype VCFs was performed. In this case, any variants shared by more than one group is dubious, as heterozygosity should be accounted for in the assembly since both haplotypes are represented. In this case, any shared variants between the groups are removed. SnpEff v5.1d (80) was used to evaluate the effect of mutations.

To classify de novo structural variation, the three svVCFs were assessed for shared variants. If a variant was present in more than one sample, it was determined ancestral. If it was only present in a single sample, it is considered de novo.

### Generating Somatic Mutation Phylogeny.

Phylogenies were constructed from de novo somatic SBS and InDels using the R packages ape (81), phangorn (82), ggtree (83), and polymorphology2 (https://github.com/greymonroe/polymorphology2). Mutations were filtered for sites in regions with a mappability score of 1, a median quality score ≧30, and a median depth ≧15. Distance between individuals was calculated using the JC69 model (84) and clustering was done using the unweighted pair group method with arithmetic mean (85). The phylogenies were bootstrapped 10,000 times and branch lengths were made proportional to the number of unique mutations in a branch.

### De Novo SBS Tricontext.

Previously identified de novo SBS were filtered for a median site quality ≧30, median depth ≧15, and a mappability score of 1. The number of SBS occurring in each context were counted, then corrected by the number of times the trimer occurred in the reference.

### Counting Telomeric Repeats.

To identify telomeric repeats in the samples, the reads aligning to the nuclear chromosomes from the bam files previously generated were extracted for each sample using SAMtools v1.13. 21-mers were counted in the nuclear reads using jellyfish v2.2.10. The counts for the telomeric repeat (TTTAGGG) occurring three times in a row were extracted and divided by the total number of base pairs in the nuclear reads for the sample.

### Assessing Allele Frequency in Aneuploids.

The ancestral sites that were previously phased were used for assessing allele frequency. Each chromosome was separated into 250 equally sized windows, and the mean allele frequency of each window was calculated. The sum of all haplotype A and haplotype B depths in each window was also calculated. A chi-square test was performed to determine if the sum of haplotype A and B depths differed significantly from the expectation of 0.5 and 0.5, the expected proportion of depths at a heterozygous site in a diploid.

### Comparing Expression across Chromosomes.

RNA count data were filtered for transcripts per million (TPM) > 1.0 and the median TPM was calculated across each chromosome. The mean of the median chromosome TPMs was then calculated for each sample, and the median chromosome TPM was normalized by the sample mean TPM.

### Gene-Level Differential Expression.

Somatic embryo samples with RNA evidence were investigated for differential RNA expression. The TPM of each transcript in every clade was compared to the TPM of that transcript in embryos that were not within the clade using a Welch’s *t* test (two-sided). Resulting *P*-values were corrected using the false discovery rate correction and adjusted *P*-values < 0.05 were considered significant. All adjusted *P*-values were −log10 transformed.

### Calling SVs.

The sorted and indexed BAM files generated from aligning long reads to the concatenated haplotype assembly with pbmm2 were used to call SVs using pbsv v2.9.0 (https://github.com/PacificBiosciences/pbsv) and generate structural variant VCFs (svVCFs).

### De Novo Structural Variation.

To identify de novo structural variation, the three samples with svVCFs generated (Embryo HiFi, Reference, Tree) were filtered for SVs that only existed in a single sample.

### Identifying Transposition and Origin.

Using Nucleotide BLAST v2.15.0+ (86), all de novo SVs detected were used as query sequences against the concatenated haplotype-phased reference assembly. Sequences were then assessed for origin location found in the haplotype-phased assembly. The SV sequences were grouped based on insertion length and origin location into two main categories, 900 class and 5,500 class variants. The first and last half of a 1,792 bp insertion matched the 900 bp class origin location, indicating an insertion followed by a tandem duplication. The sequences within classes were aligned to one another using MUSCLE (87) within the R package msa (88). The alignments were visually inspected with Geneious Prime 2022.2.1 (https://www.geneious.com) and manually trimmed to remove the target site duplication sequence incorporated during transposition (89). Using Geneious Prime, the necessary sequences were reverse complemented and all sequences were realigned using Multiple Alignment using Fast Fourier Transform (90) (*SI Appendix*, Figs. S15–S18).

### Comparing Observed Insertions to Random.

Each insertion within the 900 bp and 5,500 bp classes was assessed for its location relative to features annotated within the general feature format file. The insertions were annotated for being within gene bodies, 5′ untranslated regions (UTRs), 3′ UTRs, exons, introns, intergenic space, upstream regions, and downstream regions. Upstream regions were defined as sequence 1,000 bp before the annotated gene start and downstream regions were defined as 1,000 bp after annotated gene stop. Each insertion was then simulated to land at a random position in the target chromosome 10,000 times and the simulated insertions were assessed for their location relative to annotated features. The mean of the simulated distribution was compared to the mean of the observed data, to calculate significance.

### Proportion of Cells Containing Transposons.

Windows were created from allele frequency of 0 to 1 in increments of 0.05. The proportion of 900 bp class and 5,500 bp class insertions was assessed in each window.

### Fixation of SBS and InDels.

SBS and InDels called from the parsed haplotype-phased VCFs were further annotated for variants that were unique to a single sample. The VCFs were filtered for sites with quality values ≧5 and depth ≧10. For each sample, histograms were generated to display the occurrences of variant allele frequencies at quality scores 5 to 40 in increments of 1. The histograms of different quality thresholds were plotted on top of one another at an alpha value of 0.1.

### Disclosure of Generative AI Use.

The authors declare the use of generative AI in the research and writing process. According to the GAIDeT taxonomy (2025), generative AI was used to assist with the following tasks under full human supervision:

1. Code generation
2. Code optimization
3. Proofreading and editing

The generative artificial intelligence (GAI) tool used was ChatGPT-5.2. Responsibility for the final manuscript lies entirely with the authors. GAI tools are not listed as authors and do not bear responsibility for the final outcomes.

Generative AI was used to assist with code optimization and streamlining, as well as help identify potential for rephrasing to improve the clarity of specific sentences. All outputs were reviewed and edited.

## Supplementary Material

Appendix 01 (PDF)

Dataset S01 (XLSX)

Dataset S02 (XLSX)

Dataset S03 (XLSX)

Dataset S04 (XLSX)

Dataset S05 (XLSX)

Dataset S06 (XLSX)

Dataset S07 (XLSX)

Dataset S08 (XLSX)

Dataset S09 (XLSX)

Dataset S10 (XLSX)

Dataset S11 (XLSX)

Dataset S12 (XLSX)

## Data Availability

DNA sequencing (91–94), RNA sequencing (91), assemblies (92–94), code (95), files (96), and data (91–96) have been deposited in NCBI, GitHub, FigShare [PRJNA1271128 (91), BioProject 1289367 (92), BioProject 1289368 (93), BioProject 1289370 (94), https://github.com/matthewwdavis/Walnut-Mutation-Accumulation (95), and https://figshare.com/projects/Walnut_clones/252110 (96)]. All other data are included in the manuscript and/or supporting information.
