# Supplementary material for: Genome degradation in plant tissue culture
Source: Proc Natl Acad Sci U S A. 2026 Apr 22;123(17):e2530182123. doi: 10.1073/pnas.2530182123 (PMC13123843; doi:10.1073/pnas.2530182123)
Supplement: Supplementary file 1 — Appendix 01 (PDF) [file pnas.2530182123.sapp.pdf]

PNAS

Supporting Information for
Genome degradation in plant tissue culture

Matthew W. Davis[1*], Charles A. Leslie[1], Chaehee Lee[1], Evan Long[2], Li Meinhold[1], Megan Lorenc[1], Franklin Lewis[1], Patrick J. Brown[1*], & J. Grey Monroe[1*]

Matthew W. Davis, Patrick J Brown, J. Grey Monroe
Email: mtdavis@ucdavis.edu, pjbrown@ucdavis.edu, gmonroe@ucdavis.edu

This PDF file includes:

Supporting Information Materials and Methods
Figures S1 to S24
Supporting Information References

Other supporting materials for this manuscript include the following:

Datasets S1 to S12

Supporting Information Materials and Methods

Somatic embryo initiation & maintenance

A repetitively embryogenic somatic embryo culture derived from anther tapetal tissue of *Juglans regia* cultivar 'Chandler'(1) was maintained on corrected Driver and Kuniyuki Walnut (DKW-C) medium(2) without growth regulators, supplemented with 30g/L sucrose, and solidified with gellan gum. Cultures were kept on Petri plates continuously in the dark at room temperature. As new embryos developed by direct embryogenesis(3), a subset of embryos was transferred to fresh medium approximately weekly since 1995.

Shoot culture introduction & maintenance

Microshoot cultures of 'Chandler' were established by nodal cuttings taken from the most original field-grown source in 1985, 2010, 2013, and 2022(4). The 2010, 2013, and 2022 cuttings were generated from the same tree that provided material for the new reference assembly. Cultures were maintained under 16 hour light on DKW-C medium supplemented with 1 mg/L BAP and .01 mg/L K-IBA(2) with transfer to fresh medium every 2-4 weeks(5, 6). Recent work in strawberry suggests increased hormone levels associated with micropropagation led to higher rates of mutation(7).

DNA and RNA extraction

One somatic embryo from each subpopulation (12 in total) was selected at random and divided in half. DNA was extracted from one of the halves using the DNeasy Plant Mini Kit (QIAGEN) and RNA was extracted from the remaining half using the RNeasy Plant Mini Kit (QIAGEN). DNA was also extracted from two randomly selected embryos from the same subpopulation with the same kit. Two embryos from the same subpopulation located next to one another were pooled and nuclei were extracted using the Circulomics Plant Nuclei Protocol. Pooling was done to meet minimum tissue requirements for extraction and reduce variability, as the adjacent embryos from the same subpopulation are likely derived from the same progenitor. High molecular weight DNA was extracted from the nuclei using the Nanobind HMW DNA Extraction - Plant Nuclei kit from Circulomics. Shoot apical meristem, stem, leaf, and petiole tissue were collected from each of the shoot cultures and DNA was extracted using the DNeasy Plant Mini Kit (QIAGEN). Leaf tissue was collected from across the canopy of the two field-grown trees. Nuclei were isolated from both samples using the Circulomics Plant Nuclei Protocol, and high molecular weight DNA was extracted using the Nanobind HMW DNA Extraction - Plant Nuclei kit from Circulomics.

Library prep and sequencing

Library preparation and sequencing was conducted by the UC Davis Genome Center DNA Technologies and Expression Analysis Core Facility. Illumina Whole Genome Sequencing (150 bp, paired-end) was performed using the Illumina NovaSeq S4 300 platform. Poly-A RNA sequencing (150 bp, paired-end) was conducted using the Illumina NovaSeq X 25B platform. High molecular weight DNA was sequenced on the PacBio Sequel II platform (Dataset S1).

Omni-C data generation

Leaf tissue from across the canopy of the Reference clone was collected, pooled into a 50 ml tube, and flash frozen with liquid nitrogen. The sample was shipped to Cantata Bio for Omni-C library preparation and sequencing. The Omni-C library was prepared using the Dovetail®

Omni-C® Kit according to the manufacturer's protocol. Briefly, the chromatin was fixed with disuccinimidyl glutarate (DSG) and formaldehyde in the nucleus. The cross-linked chromatin was then digested in situ with DNase I. Following digestion, the cells were lysed with SDS to extract the chromatin fragments and the chromatin fragments were bound to Chromatin Capture Beads. Next, the chromatin ends were repaired and ligated to a biotinylated bridge adapter followed by proximity ligation of adapter-containing ends. After proximity ligation, the crosslinks were reversed, the associated proteins were degraded, and the DNA was purified then converted into a sequencing library using Illumina-compatible adaptors. Biotin-containing fragments were isolated using streptavidin beads prior to PCR amplification. The library was sequenced on an Illumina HiSeq X platform to generate 400 million 2 x 150 bp read pairs.

Reference genome assembly

To allow better mutation detection(8, 9), a haplotype-phased reference genome assembly was constructed using Hifiasm v0.19.8(10–12) from the HiFi CCS reads and Omni-C reads generated for the field grown Reference clone. The primary and phased contigs generated by Hifiasm were then scaffolded using the previous Oxford Nanopore Technologies, Hi-C, and short read generated walnut cultivar 'Chandler' genome ('Chandler' v2.0) with RagTag v2.1.0(13).

A 'Chandler' genetic map was constructed using genotyping-by-sequencing data from 436 self-pollinated 'Chandler' progeny. Data were imputed using FSFHap(14), resulting in a dataset of 16,282 SNPs. One individual with >5% missing data was discarded. SNPs were filtered to retain only those with 30-70% heterozygous genotypes and <5% missing data, resulting in the removal of 37 SNPs. Testing for segregation distortion of genotypes and alleles revealed no remaining SNPs with chi-square p-values below 0.001. The ASMap package in R(15) was used to construct a genetic map of 16,245 markers and 3,660 bins and near-perfect agreement in marker order between genetic and physical maps (SI Appendix, Fig. S6). The genetic map was then used to verify phasing in the reference genome by assessing the accuracy of the haplotype assignment by Hifiasm by identifying haplotype and primary assembly correspondence with SyRI v1.5.4(16) and using custom R scripts (SI Appendix, Fig. S1*B*).

The assembled haplotypes were concatenated into a single fasta file, creating four assemblies: The primary assembly, haplotype A assembly, haplotype B assembly, and a concatenated haplotype A and B assembly.

Omni-C reads were then aligned to the primary, haplotype A, and haplotype B assemblies and contact matrices were generated using the Dovetail Genomics protocol (https://omni-c.readthedocs.io/en/latest/contact_map.html) and JuicerTools(17) (SI Appendix, Fig. S2). Telomere presence was assessed using the R package ggenomics (https://github.com/matthewwdavis/ggenomics).

Comparing Assemblies

Chromosome–scale assemblies were aligned using Minimap2 v2.17-r941(18, 19) and structural variation between the assemblies was assessed using SyRI and visualized using plotSR v0.5.4(20). Structural similarity was also assessed between the assemblies using the Minimap2 generated alignments and dot plots generated by the D-GENIES(21) browser tool (SI Appendix, Fig. S4).

Annotating the genome assemblies

For the primary, haplotype A, and haplotype B genome assemblies, annotations were generated using Liftoff v1.6.3(22) and the 'Chandler' v2.0 GFF(23).

Transposable elements (TEs) were annotated on the primary assembly using The Extensive *de novo* TE Annotator (EDTA) v2.1.0(24) with default parameters. We removed transposable elements that overlapped with the filtered plant protein database using protExcluder v1.2 (https://www.canr.msu.edu/hrt/uploads/535/78637/ProtExcluder1.2.tar.gz) to reduce the exclusion of genes in any further gene prediction. The TEs were re-annotated on the primary contig assembly using a filtered, non-redundant TE library, and the assembly was softmasked.

The annotations were further processed for downstream analysis in R v.4.4.2(25) with custom scripts. Annotated genes and CDS were extracted, transcribed, and translated. Genes and CDS that did not begin with a start codon (AUG) and end with stop codons (UAA, UAG, UGA) were removed. Intergenic space, 5' UTRs, 3' UTRs, and introns were annotated with custom R scripts.

Assessing genome assemblies

To assess assembly completeness, BUSCO v5.7.1(26) analysis was performed using the eudicots_odb10 database. Reference tree reads were assessed for 21-mer content using jellyfish v2.2.10(27) and the GenomeScope v2.0(28) web browser tool was used to assess the kmer distribution. The annotated genes in the GFF were used to determine the proportion of the genome constituting gene bodies (SI Appendix, Fig. S3). Assembly statistics were evaluated using SeqKit v2.10.0(29) (SI Appendix, Fig. S1, Dataset S2).

Genome mappability

Mappability was assessed using GenMap v1.3.0(30), specifying a kmer length of 150 and allowing for 0 errors. When annotating regions as mappable using custom R scripts, we retained regions with a mapping probability of 1.

Verifying samples are clonal

SBS and small InDels were called against the 'Chandler' primary assembly using WGS data of 5 other *Juglans regia* cultivars ('Franquette', 'Hartley', 'Payne', 'PI159568', & 'Waterloo'). A SBS PCA was generated using R to visualize the relationship between the clones and the other cultivars, as well as the relationship of the clones to each other. (SI Appendix, Fig. S24).

Mapping RNA sequencing data

RNA sequencing data was pseudo-aligned to the primary reference assembly and counts were generated and normalized to transcripts per million (TPM) using kallisto 0.51.0(31).

Layer specific *de novo* SBS tricontext

Previously identified *de novo* SBS from the combined haplotypes were filtered for a median site quality >=20 and a median depth >=5. The number of SBS occurring in each context were counted, then corrected by the number of times the trimer occurred in the reference. Mutations with a frequency < 0.5 were determined L1, and mutations with a frequency > 0.5 were determined to be L2/L3.

Similarity of spectra to known profiles

Known SBS profiles were retrieved from COSMIC(32), and the similarity to each spectra was calculated using the cosine similarity.

$$\cos(\theta) = \frac{\sum(x*y)}{\sqrt{\sum(x^2)} * \sqrt{\sum(y^2)}}$$

Identifying mutation location

Mutations defined as *de novo* with a median site quality >=30, median depth >=15, and a mappability score of 1 were assessed for overlap with the curated list of genes using custom R scripts and the polymorphology2 package.

Calculating per year mutation rates

To estimate the per year mutation rate in plants, several sources of available reports and data were used(33–36). If the mutation rate was not explicitly specified, it was calculated by dividing the reported number of mutations by the estimated genome size, then dividing the quotient by the reported number of years. To calculate the mammalian per year somatic mutation rates from available data(37), the number of SBS and the number of InDels in each sample were divided by the genome size, and the quotient was then divided by the age of the sample. The species somatic mutation rate was then determined as the average of all sample rates.

Calculating scaled depth in chromosomes

Genomic instability can have dramatic effects on phenotype(38, 39). All sites previously identified as ancestral heterozygosity when aligned to the primary reference genome were assigned haplotypes using the genetic map. The alternate call was used to represent one haplotype, with the allele frequency and alternate read depth associated with it. The other haplotype was then represented by the reference call, with one minus the allele frequency representing the frequency at that site and the reference depth representing the read depth. The depth for each site was scaled by the mean depth of the sample. Each chromosome was separated into 7 equally sized segments, and the mean of the scaled depth was taken at each segment. Haplotype assigned scaled read depth was also used without windows to plot chromosome duplications, somatic recombinations, and large deletions (SI Appendix, Fig. S11*A*).

Comparing telomeric repeat numbers

The telomeric repeat counts of all embryos were compared to the counts of shoots and trees with a Welch's t-test (two-sided) using custom R scripts. Embryos were then separated into the two major clades and compared to one another with a Welch's t-test (two-sided). The clade with fewer repeats was then compared to the trees and shoots in a similar manner.

Comparing TERT and TCAB1 expression

The pseudo-aligned RNA sequencing data was filtered for read counts of annotated TERT and TCAB1. Embryos were then separated into the two major clades and compared to one another with a Welch's t-test (two-sided).

Simulating ploidy changes

Each site in the VCF of the clones sequenced with PacBio HiFi was filtered for a quality >=40 and a depth >=30. The difference in sequencing depth between the depth of the reference allele and alternate allele of each site was then simulated with a weighted binomial distribution.

To simulate a diploid, the weighted depths were ½ and ½, a triploid was weighted ⅓ and ⅔, and an asymmetric tetraploid was weighted ¼ and ¾. These distributions for each chromosome were plotted, with the observed distributions of the chromosomes also plotted (SI Appendix, Fig. S12*B*).

Timing duplications

The relative timing of the chromosome 4A and 9B duplications was identified through allele frequencies. In the primary and haplotype-resolved assemblies, *de novo* mutations were filtered for those shared by all embryos and with a median quality >=30, depth >=15, and a mapping probability of 1. A chi-square test was performed to determine if the mutations differed significantly from the diploid expectation of 0.5 and 0.5. If the p-value was <0.01 and the allele frequency was >0.5, the site was determined as duplicated. If allele frequency was <0.5, it was non-duplicated. The mutations occurring on chromosomes 4 and 9 were plotted as a scatterplot to visualize. The duplicated mutations on these chromosomes were corrected for chromosome length and compared with Welch's t-test (two-sided). The ratios of the duplicated to non-duplicated mutations on these chromosomes were also compared using Welch's t-test (two-sided) (SI Appendix, Fig. S11 *C*-*E*).

Determining chromosome loss

To identify if the chromosome 4 duplication occurred multiple times independently or occurred once and was lost, *de novo* mutations in the haplotype-resolved assemblies in chromosome 4 were filtered for quality >=30. A chi-square test was performed to determine if the mutations differed significantly from the expectation of 0.5 and 0.5 and a False Discovery Rate (FDR) correction was applied. If the p-value was <0.05 and the allele frequency was >0.5, it was determined to be a mutation that occurred before duplication. If the allele frequency was <0.5 it was identified as a post-duplication mutation. The mutations that occurred post-duplication but were still found in the non-duplicated samples were identified as retained post-duplication mutations (SI Appendix, Fig. S11*B*).

Identifying transposition with short-read sequencing

We used the initially discovered 5500 class TE on chromosome 13 in the embryo sequenced with HiFi as a seed to track its activity across the genome and across samples. For each sample, we collected all reads mapping to the TE origin and then retrieved all alignments of those same reads anywhere in the genome, capturing both split and paired-end mappings. These alignments were clustered by genomic proximity (1 kb radius) on primary chromosomes to define putative TE insertion loci, and loci supported by at least 4 reads. We summarized these loci as a presence–absence matrix of TE insertions across samples and inferred a TE-based somatic tree using binary distances and UPGMA.

For each locus, we used kernel density estimation of read breakpoints to infer a precise insertion coordinate and to examine spacing between insertion sites. Insertion positions were annotated relative to genes (gene body, ±2 kb upstream/downstream), and for gene-body insertions we computed a strand-aware relative position from transcription start to termination. RNA-seq data were aggregated to gene-level expression per sample. For each gene with a neighboring or internal TE insertion, we compared expression between samples with versus without the insertion, summarizing fold-changes and mean z-scores and testing for global trends with a two-way T-test to compare observed trend against a null of 0 (no trend in expression

difference between TE vs no TE samples for that gene). This analysis was conducted using the R package kolibri (https://github.com/greymonroe/kolibri).

Identifying transposition with RNA sequencing

RNA sequencing reads were aligned to the concatenated haplotypes of the reference genome using the splice-aware aligner STAR(40). The alignment files were then queried for reads aligning to the origin locations of the 5500 and 900 class TEs.

Aligning identified TEs for consensus

Verified and trimmed TE sequences were aligned to one another using MUSCLE in the R package msa. The consensus sequence was determined as the most common nucleotide at a specific position. TE sequences with large size differences were removed from the alignment to improve legibility (SI Appendix, Fig. S20 and S21).

Assessing transposon morphology

Using Nucleotide BLAST v2.15.0+, all *de novo* SVs detected were used as query sequences against the fasta file of EDTA predicted transposable elements. Open reading frames (ORFs) in the 900 bp class and 5500 bp class of sequences were identified using NCBI ORFfinder (https://www.ncbi.nlm.nih.gov/orffinder/). Identified ORFs were searched for similarity in the NCBI database using Protein BLAST(41). Geneious Prime was used to further validate ORFs and identify repeat regions in the 900 bp and 5500 bp classes (SI Appendix, Fig. S15 and 16).

Photographs

Photographs of germinated somatic embryo shoots and shoot culture Shoot 2022 were taken on a Sony A7III. Contrast was increased to better visualize the plants.

Sample naming

Alternative names for samples used throughout various scripts and in repositories and their corresponding names in the manuscript can be found in Dataset S12.

Diagrams

Diagrams were created using Microsoft PowerPoint v16.95 and BioRender.

Figures

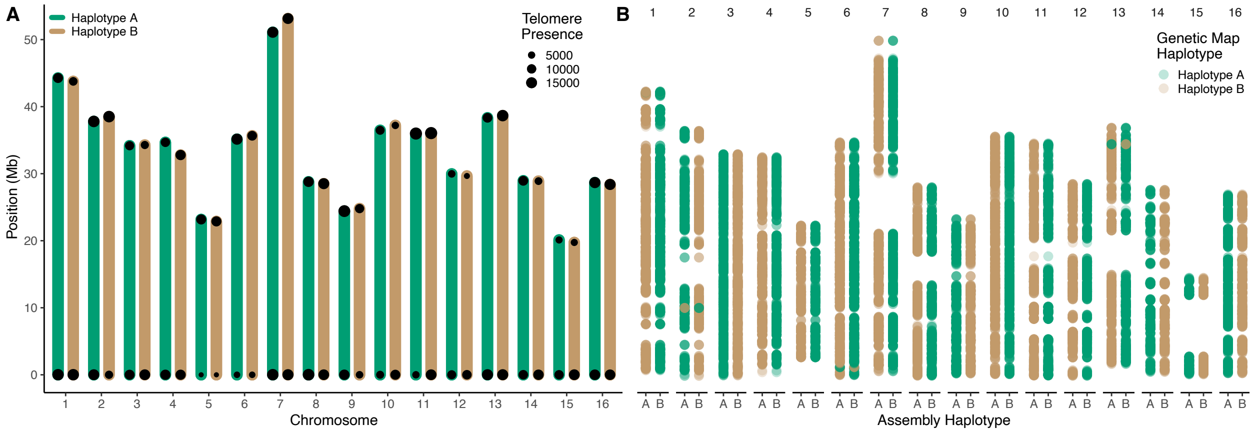

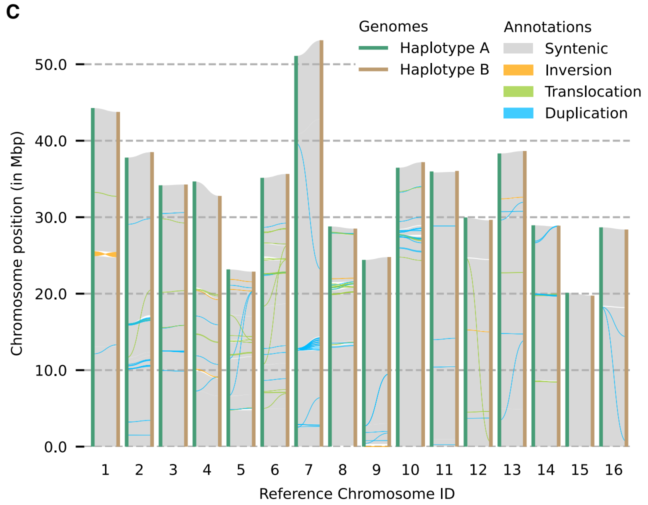

D

***Juglans regia* 'Chandler'**

	Primary	Haplotype A	Haplotype B
No. of seqs	431	356	189
No. of chromosomes	16	16	16
Total Size (bp)	563685698	549707088	547053093
Size of chromosomes (bp)	540705719	532913467	532913467
Max length (bp)	52578039	51092591	53139419
N50 (bp)	36346324	35147692	35147692
L50	7	7	7
No. of gaps	18	39	44
Avg. no. contigs per chromosome	1.125	2.4375	2.75
GC (%)	36.46	36.36	36.44
BUSCO completeness (%)	98.1	98.1	98.1
Complete single-copy (%)	90.2	90.2	90.4
Complete duplicated (%)	7.9	7.9	7.7

Fig. S1.
(**A**) Depiction of haplotype phased genome assembly. Bars represent length of chromosome, point size represents location and number of telomeric repeats. (**B**) The accuracy of haplotype phasing as determined by the genetic map. The haplotype assigned to the assembly is represented on the x axis and the color of the point corresponds to the genetic map haplotype. (**C**) Structural variation between the two haplotypes of Juglans regia 'Chandler'. (**D**) Table depicting assembly statistics.

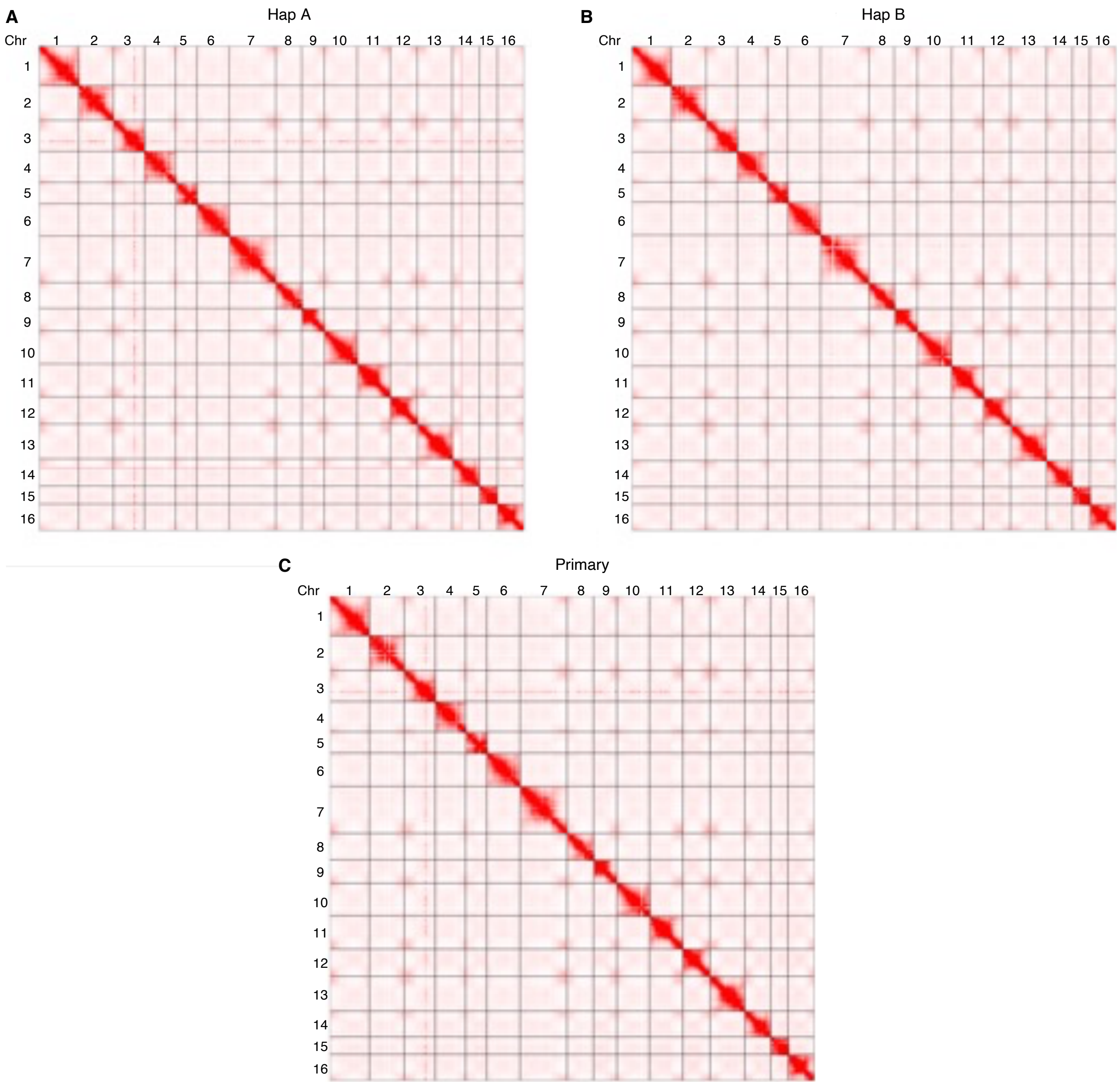

Fig. S2.
(**A**) Contact map of Omni-C reads to the haplotype A Reference tree genome assembly. (**B**) Contact map of Omni-C reads to the haplotype B Reference tree genome assembly. (**C**) Contact map of Omni-C reads to the primary Reference tree genome assembly.

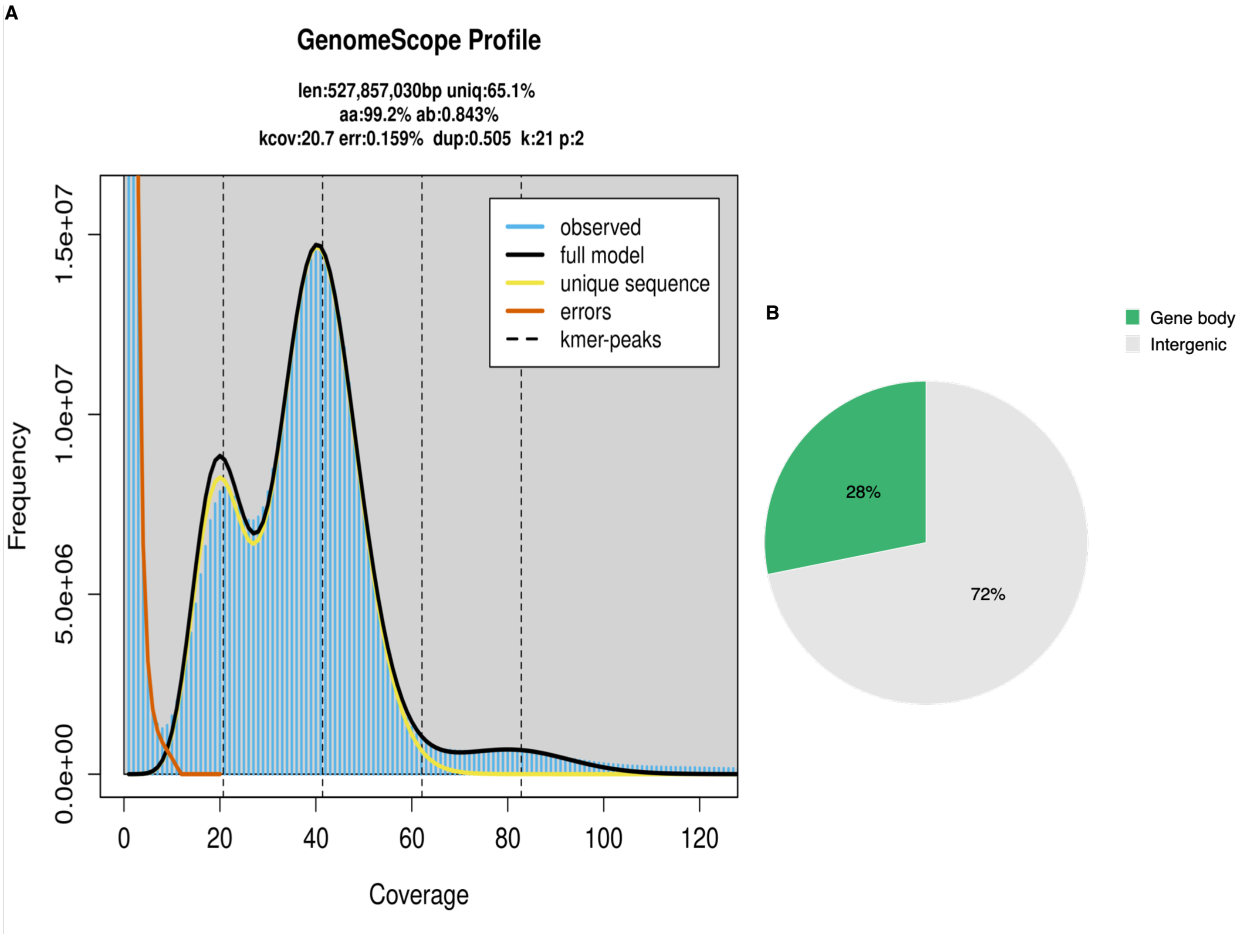

Fig. S3.
(**A**) GenomeScope profile of the reference reads. (**B**) Proportion of the genome that is annotated gene bodies compared to the rest of the genome.

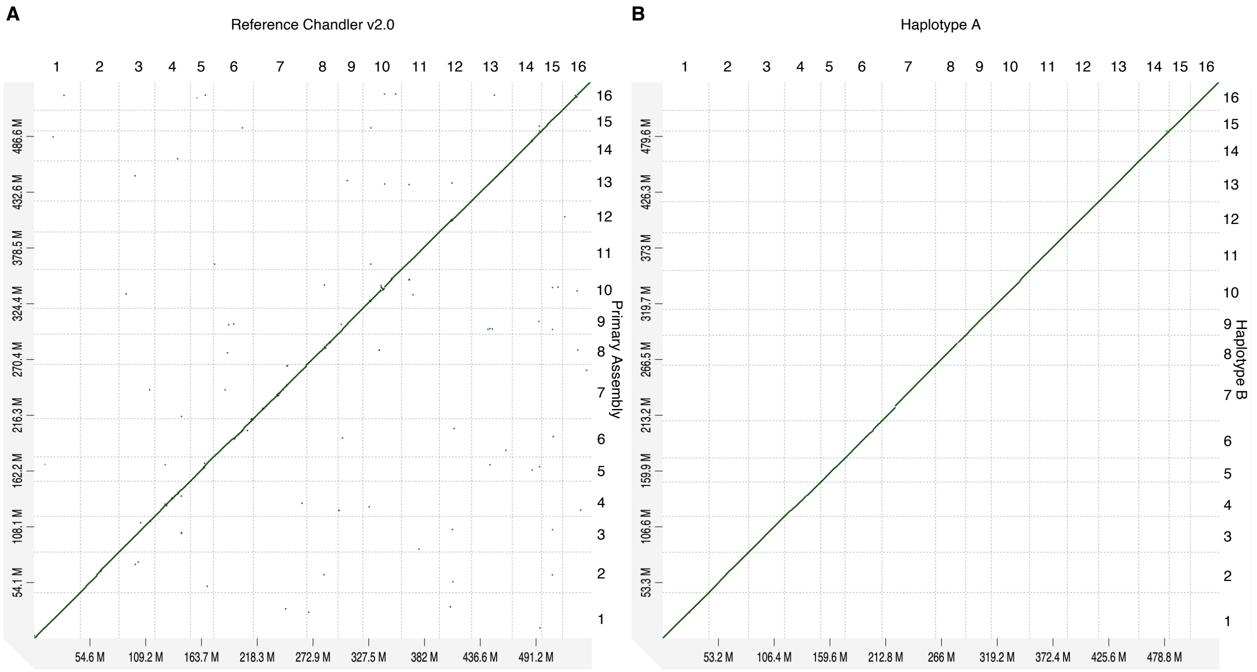

Fig. S4.
(**A**) D-Genies dot plot visualizing the alignment of the Reference Chandler V2.0 assembly and the newly constructed primary sequence. (**B**) D-Genies dot plot visualizing the alignment of the haplotype A assembly and the haplotype B assembly.

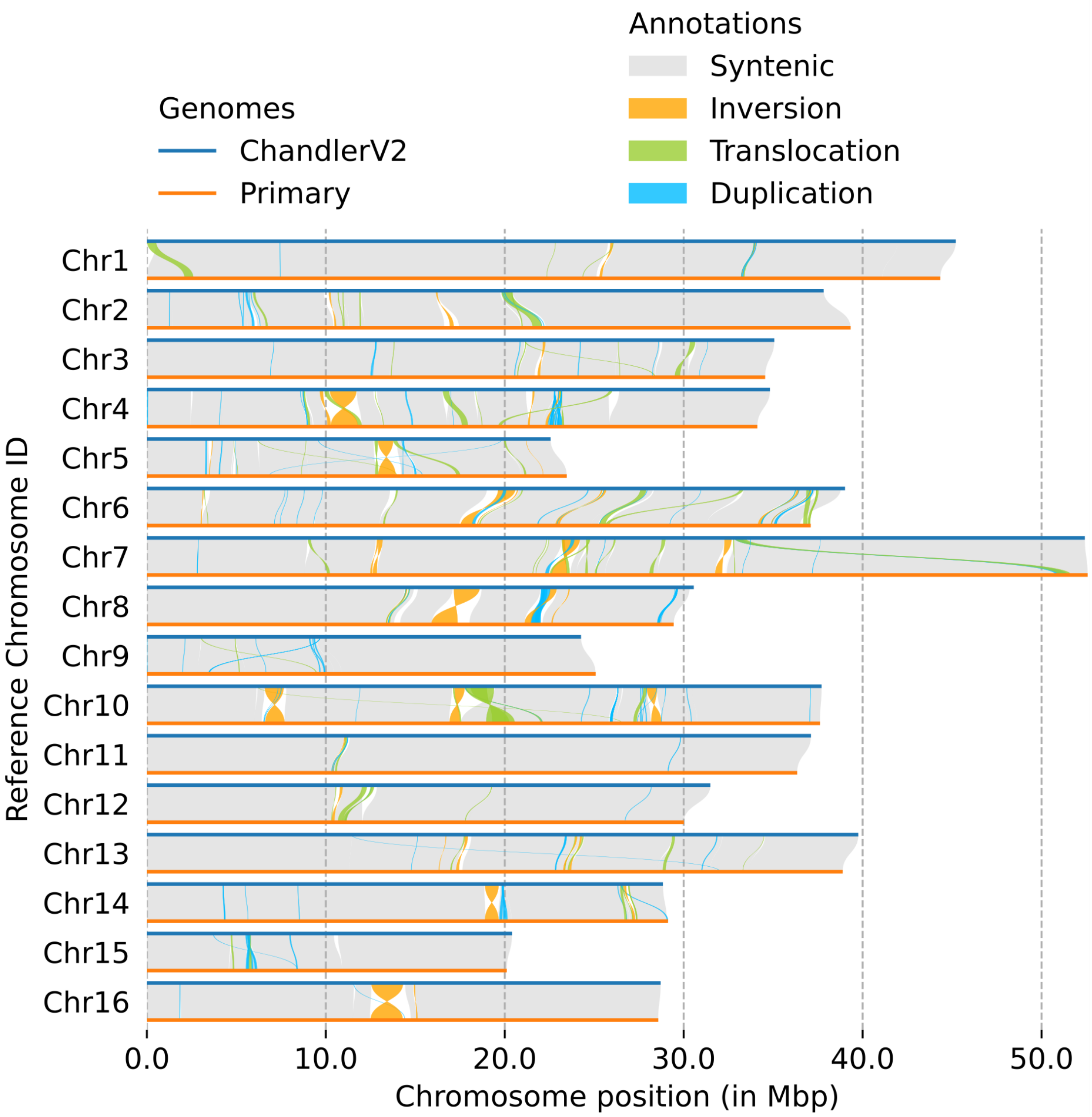

Fig. S5.
Structural variation between the Chandler v2.0 reference sequence and the primary assembly.

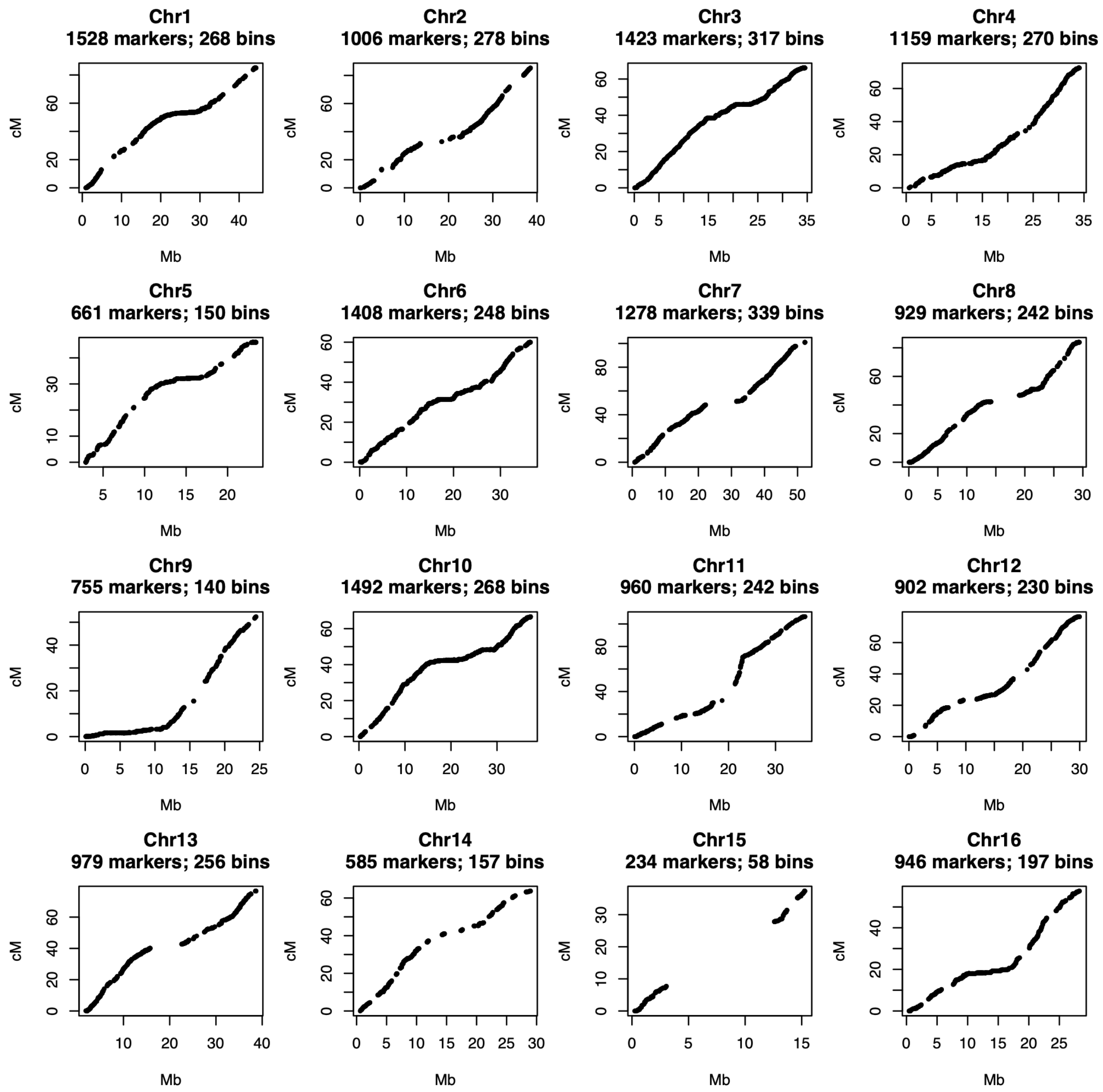

Fig. S6.
The agreement of marker order in the genetic map and physical map for the 16 chromosomes of Juglans regia ‘Chandler’.

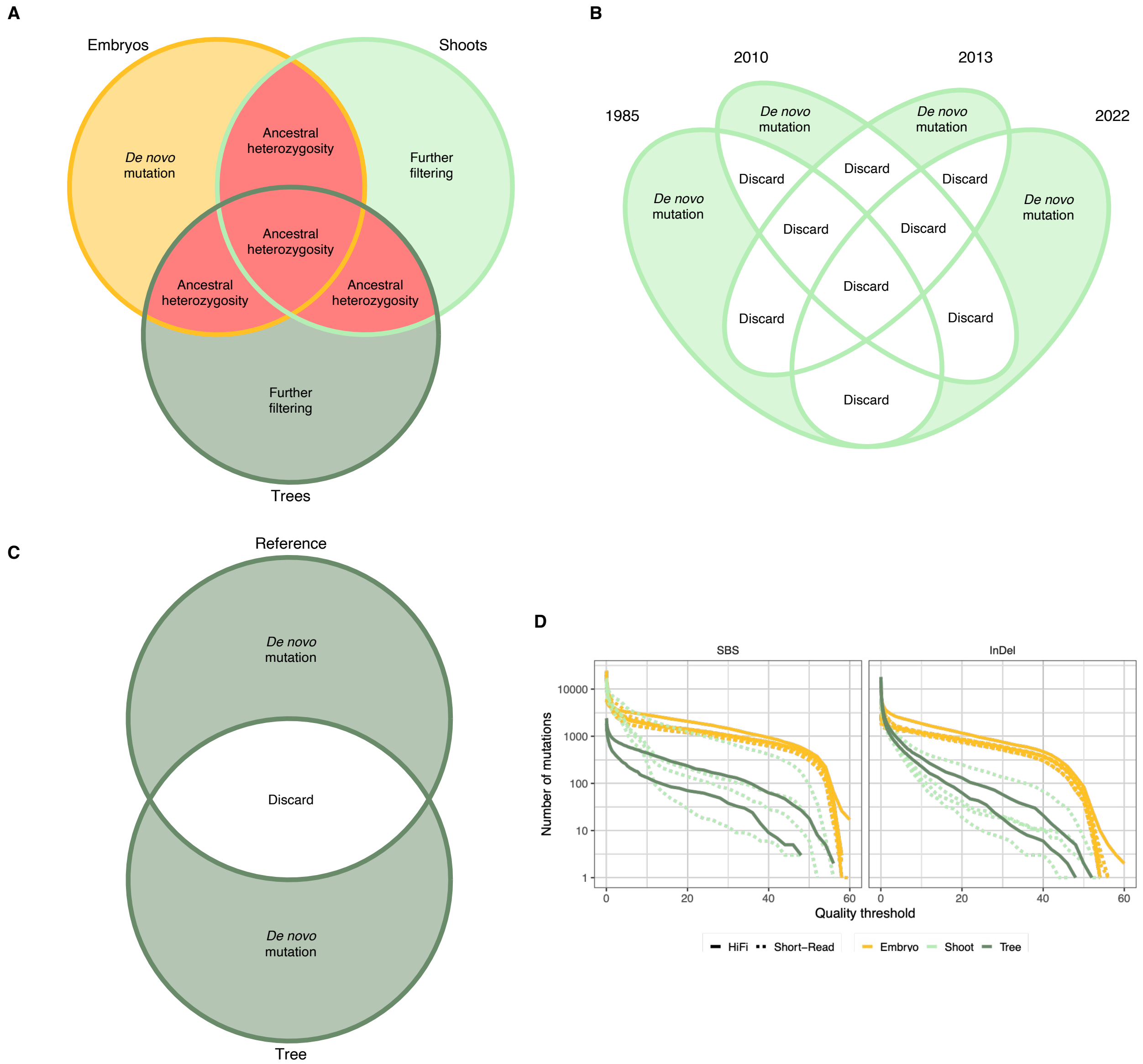

Fig. S7.
(**A**) Venn diagram depicting the determination of *de novo* mutation in the somatic embryos and ancestral heterozygosity in the clones. (**B**) Venn diagram depicting the determination of *de novo* mutation in the shoot cultures. (**C**) Venn diagram depicting the determination of *de novo* mutation in the field-grown trees. (**D**) Number of *de novo* SBS and InDels at differing filtering quality thresholds in every sample.

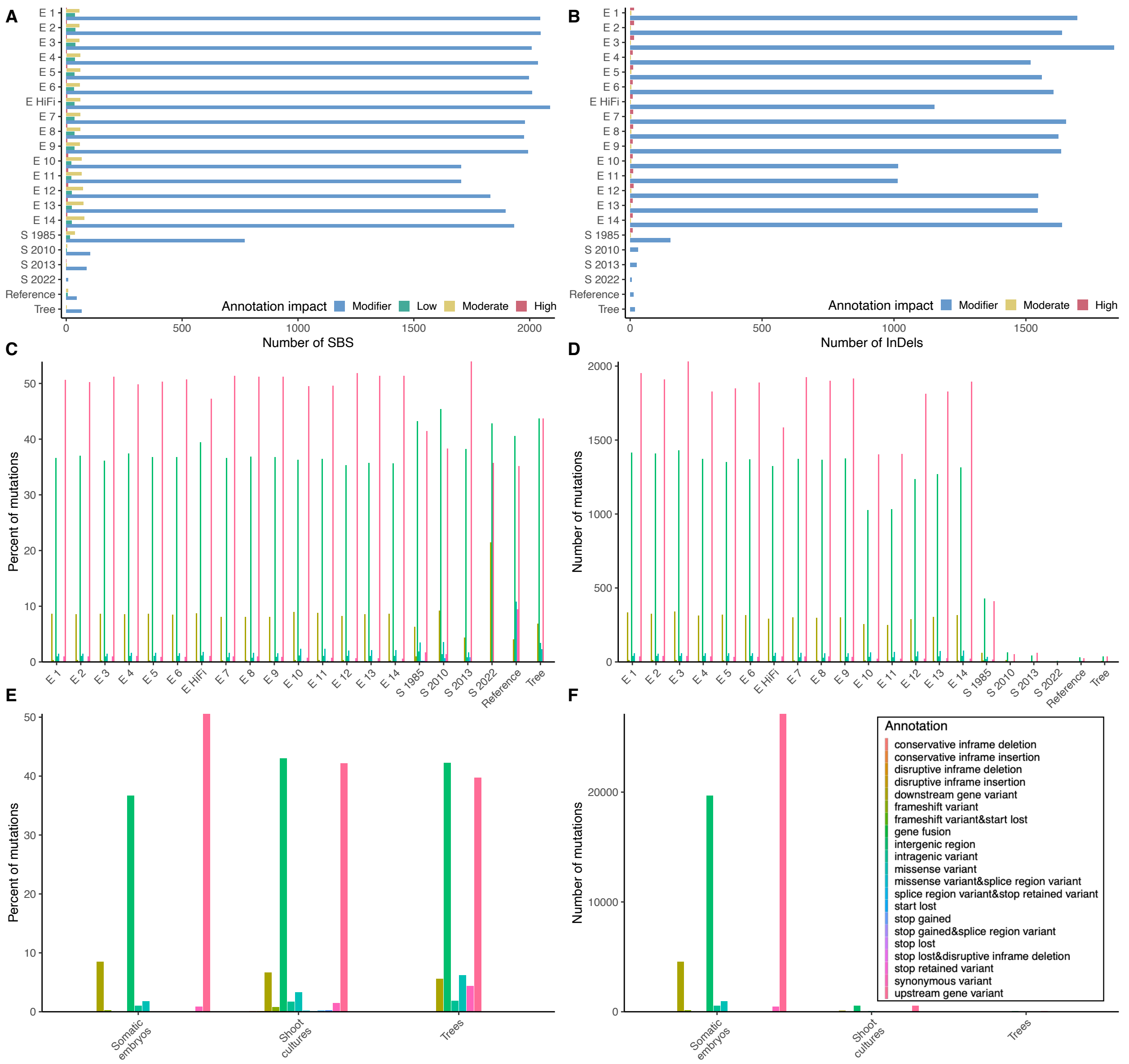

Fig. S8.
(A) The number of SBS mutations in each annotation impact category determined by SnpEff in each clone. (**B**) The number of InDel mutations in each annotation impact category determined by SnpEff in each clone. (**C**) The percent of mutations in functional classes annotated by SnpEff within each clone. Legend is shared with panel F. (**D**) The number of mutations in functional classes annotated by SnpEff within each clone. Legend is shared with panel F. (**E**) The percent of mutations in functional classes annotated by SnpEff within each clonal propagation method. Legend is shared with panel F. (**F**) The number of mutations in functional classes annotated by SnpEff within each clonal propagation method.

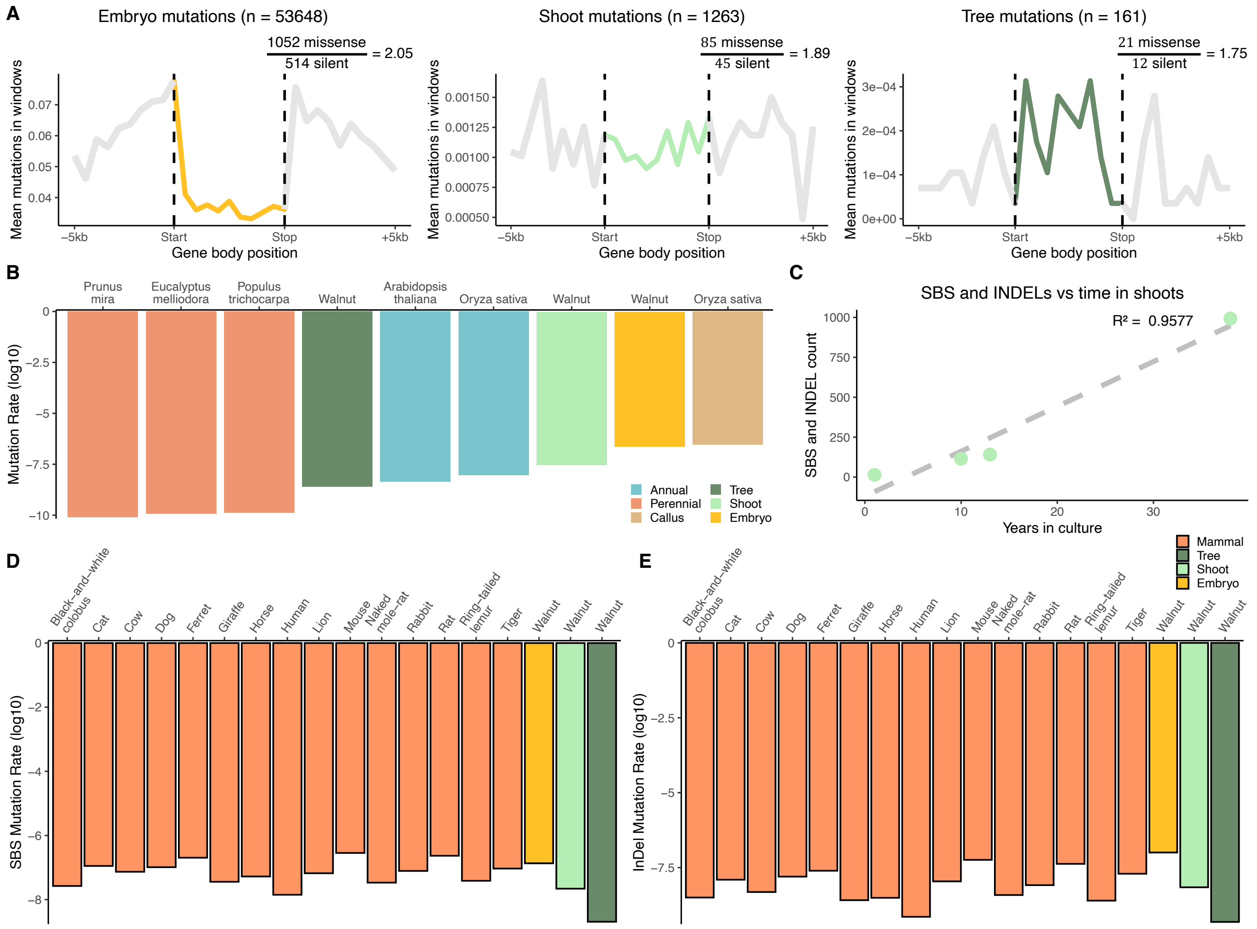

Fig. S9.
(**A**) *De novo* SBS and InDels in the gene body compared to upstream and downstream regions. Samples pooled by method of clonal propagation, n value refers to the number of total mutations. (**B**) Comparison of previously reported somatic and germline yearly mutation rates in other species and the yearly somatic mutation rates of the walnut clones. (**C**) SBS and InDels in shoot clones introduced to tissue culture at different times. The relationship between years in culture and mutation count was assessed with a linear model, and model fit was determined. (**D**) Comparison of previously reported somatic per year SBS mutation rates in other species with the per year SBS mutation rates of the walnut clones. (**E**) Comparison of previously reported somatic per year InDel mutation rates in other species with the per year InDel mutation rates of the walnut clones.

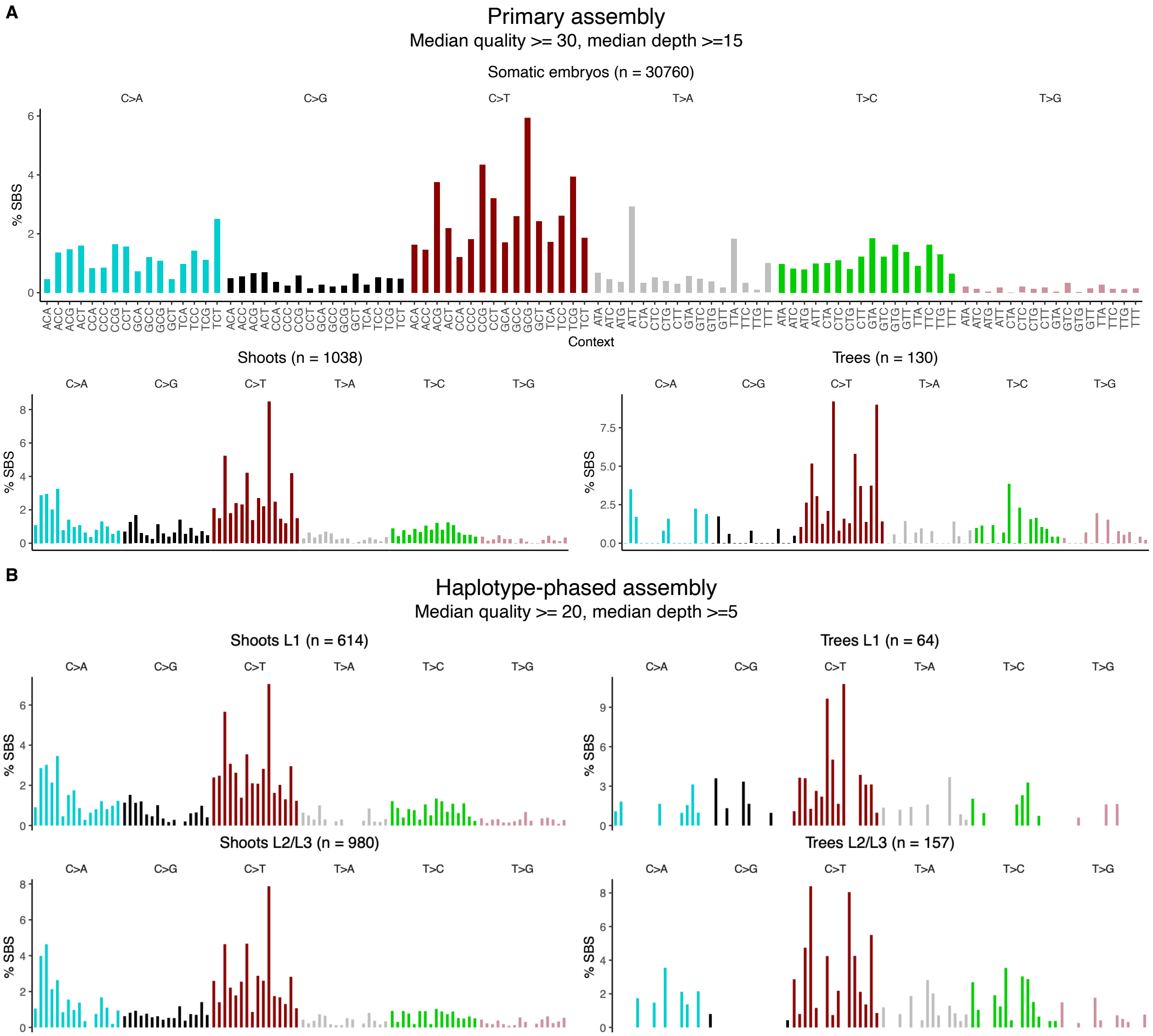

Fig. S10.

(**A**) *De novo* SBS spectrum of samples pooled by method of clonal propagation. The n value refers to the number of total mutations. (**B**) *De novo* SBS spectrum of mutations called against the haplotype-phased assembly and separated into putative L1 and L2/L3 spectra.

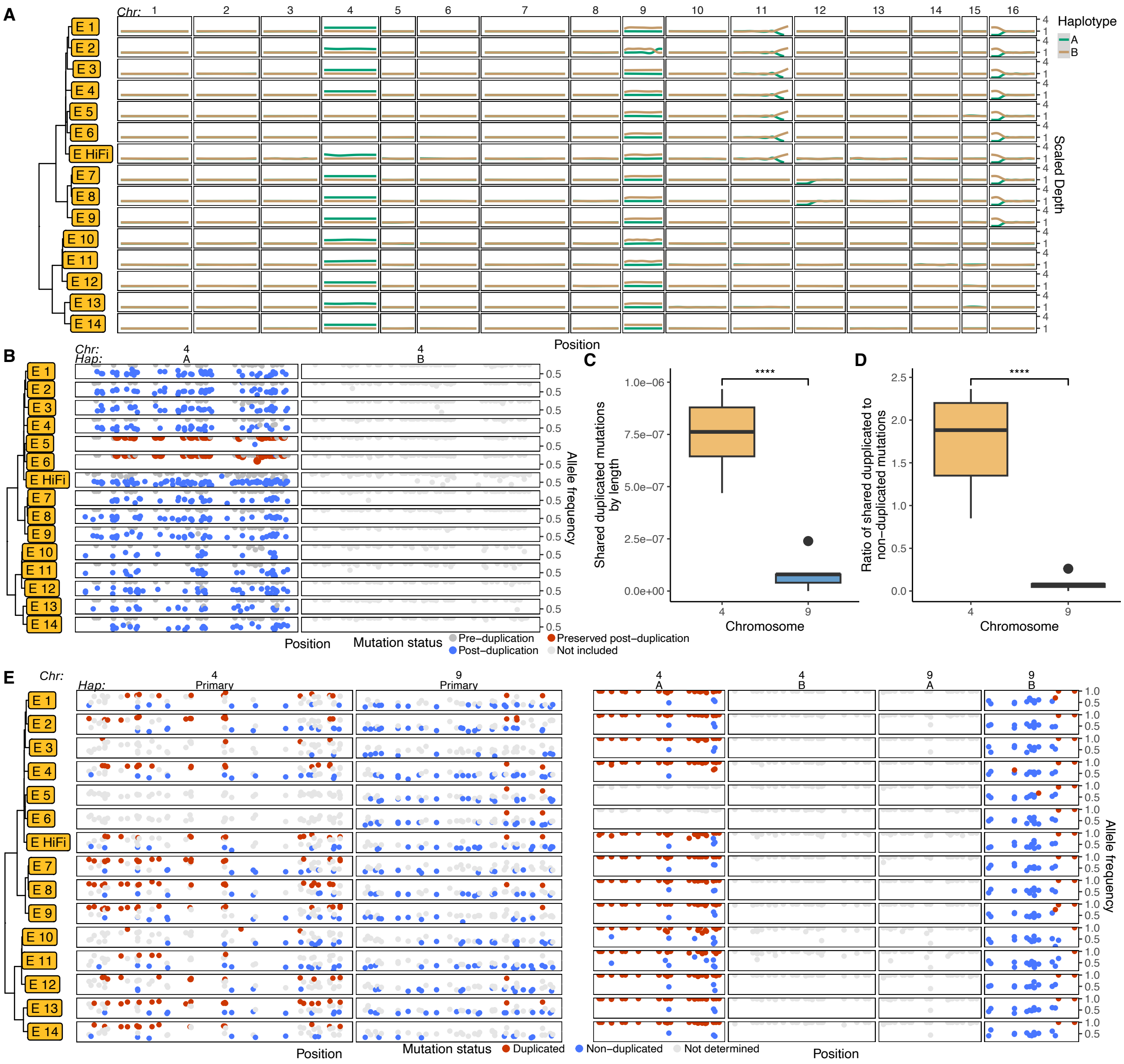

Fig. S11.

(**A**) Haplotype-resolved depth across chromosomes in the somatic embryos. (**B**) *De novo* mutations that occurred after chromosomal duplication (blue) that also exist in the non-duplicated samples (red). (**C**) The number of duplicated shared *de novo* mutations corrected by chromosome length in chromosomes 4 and 9. (**D**) The ratio of duplicated shared *de novo* mutation to non-duplicated shared de novo mutations in chromosomes 4 and 9. (**E**) Shared *de novo* mutation by all somatic embryos in the primary and haplotype-resolved assemblies. Red points denote mutations that occurred before duplication, blue points are mutations that occurred after.

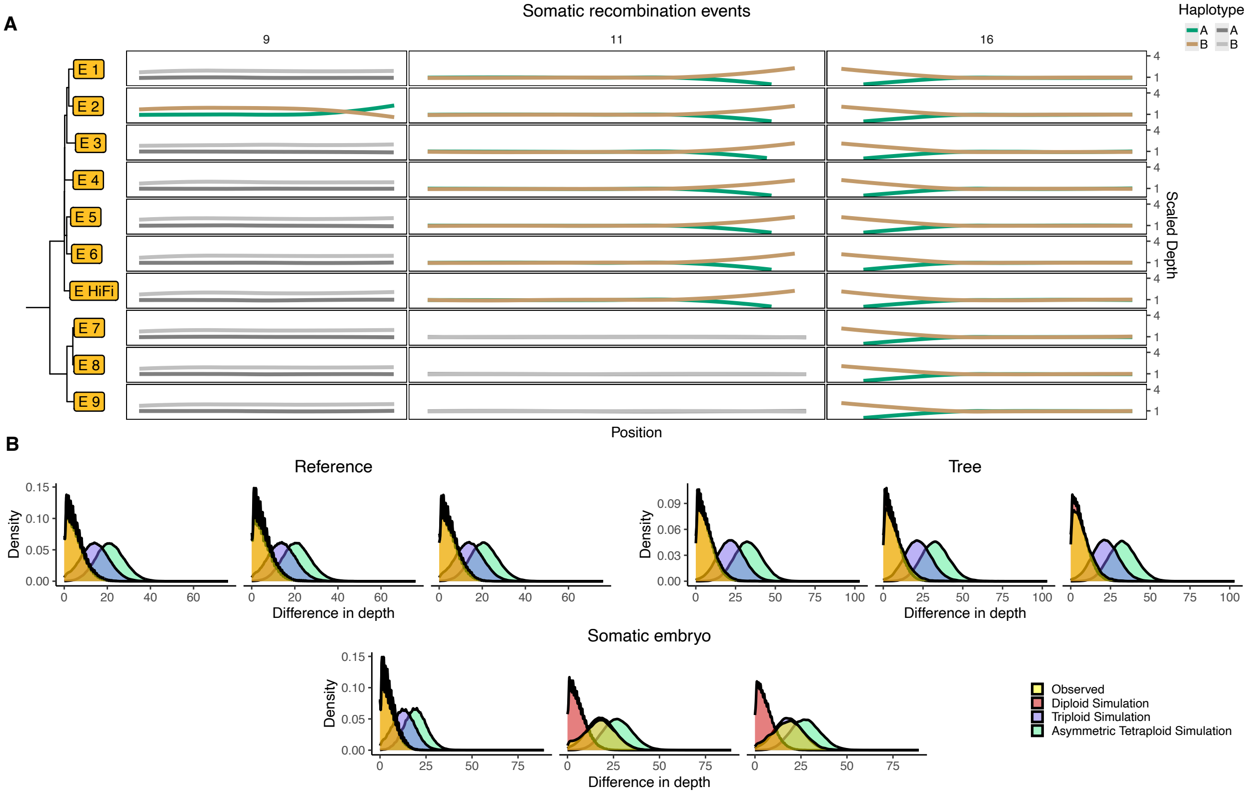

Fig. S12.

(**A**) Haplotype-resolved depth across chromosomes exhibiting somatic recombination in the somatic embryos. (**B**) For all long-read sequenced samples, the difference between the reference site depth and alternate site depth was taken. The expected differences in these depths were calculated based on proposed diploid (equal weight), triploid (1/3 and 2/3), and asymmetric tetraploid (1/4 and 3/4) ratios sampled from a binomial distribution. These simulated distributions were plotted along with the distributions of the observed data in the three long-read sequenced samples. The chromosomes duplicated in the somatic embryos are shown in the somatic embryo and tree clones, as well as chromosome one to represent a typical diploid chromosome.

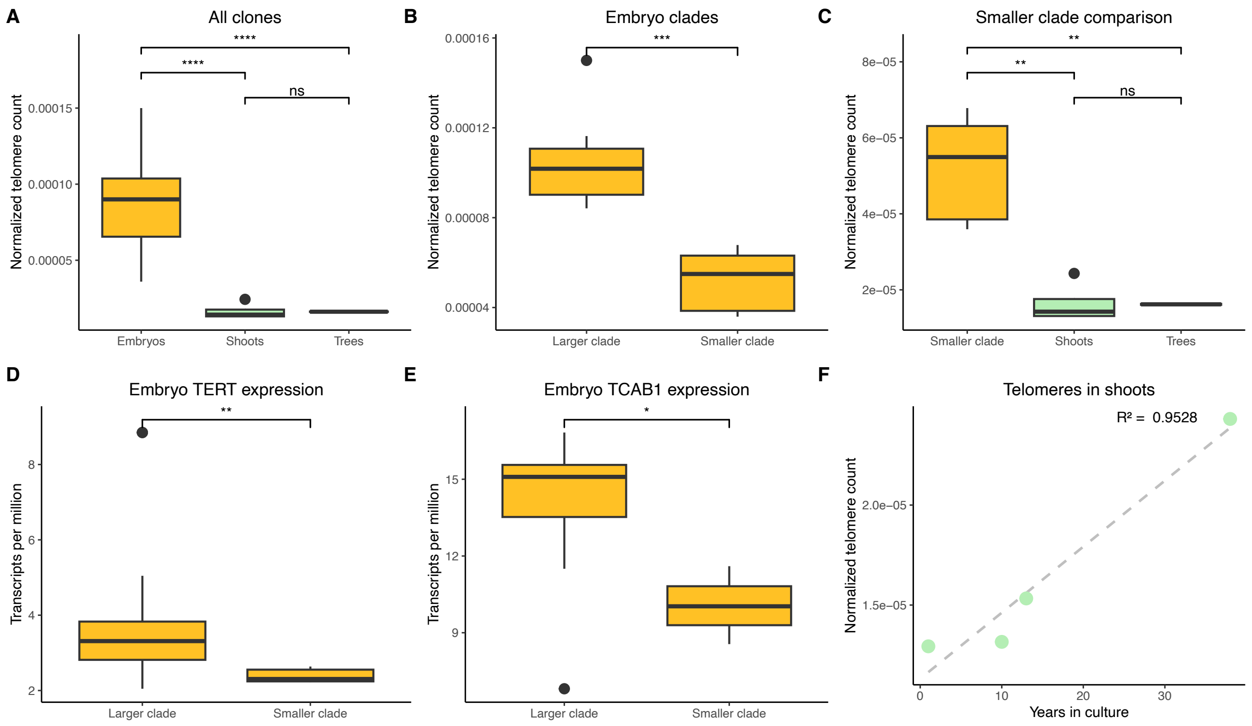

Fig. S13.
(**A**) Number of telomeric repeats pooled by method of clonal propagation. A Welch's t-test was performed comparing the somatic embryos to the shoots and trees. ****: p <= 0.0001, ***: p <= 0.001, **: p <= 0.01, *: p <= 0.05. (**B**) Somatic embryos were separated into the two largest clades. The telomeric repeats in each clade were pooled and compared to one another. A Welch's t-test was performed comparing the two clades. (**C**) The telomere repeats of the smaller of the two somatic embryo clades were pooled and compared to the shoots and trees. (**D**) The expression of TERT compared between the larger and smaller embryo clades. (**E**) . The expression of TCAB1 compared between the larger and smaller embryo clades. (**F**) The telomeric repeats in the shoot clones. The relationship between years in culture and telomeric repeat count was assessed with a linear model, and model fit was determined.

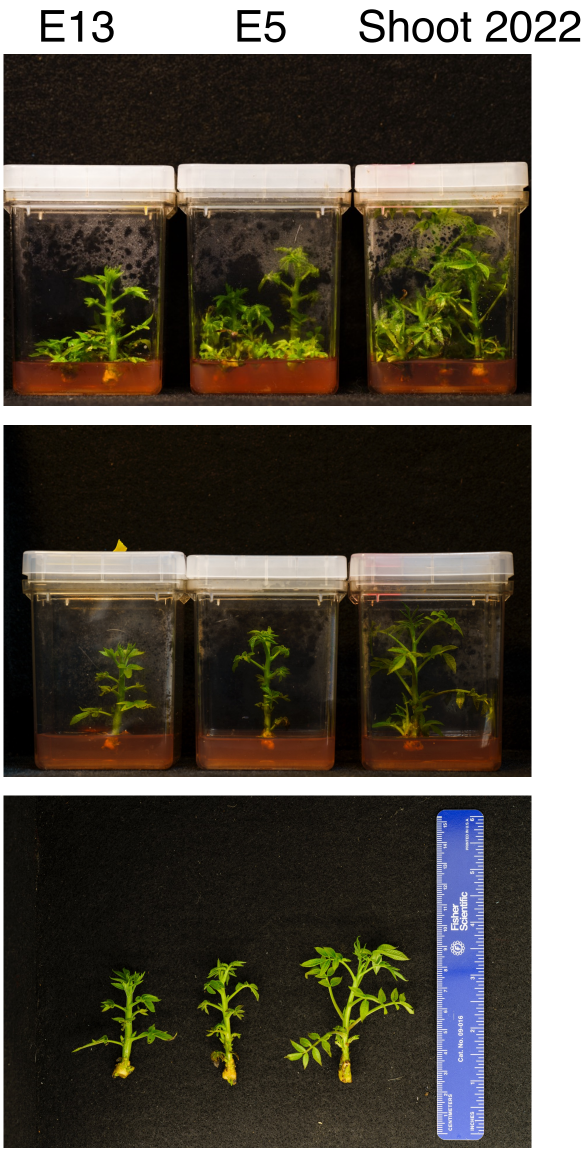

Fig. S14.
Photos of induced shoots from somatic embryos E13 and E5, as well as the shoot culture Shoot 2022. The shoots induced from the somatic embryos are less vigorous and have delayed growth, despite being transferred two days earlier than the Shoot 2022 culture. They also display shortened internodes and more compact leaf morphology than the Shoot 2022 culture.

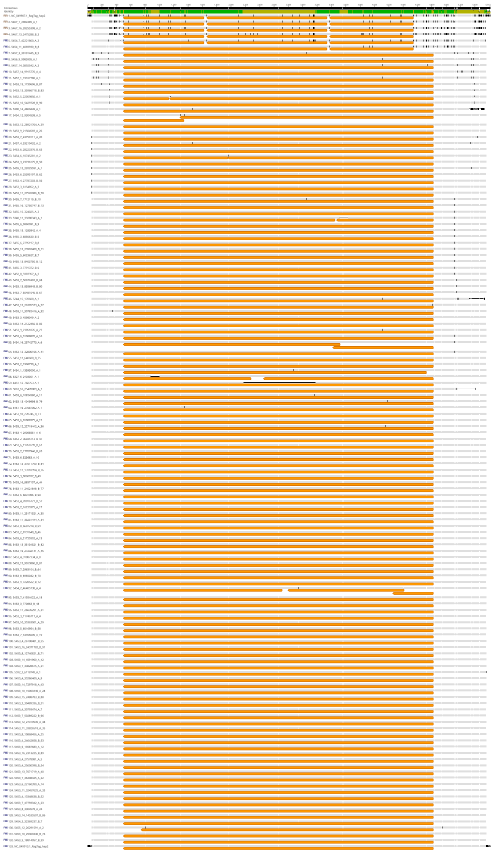

Fig. S15.
Alignment of all trimmed 5500 class TEs and the matching sequences from the reference genome assembly visualized with Geneious. Open reading frames are annotated in orange boxes underneath the sequences. The sequences matching consensus are represented in grey, with alternate bases displayed in black.

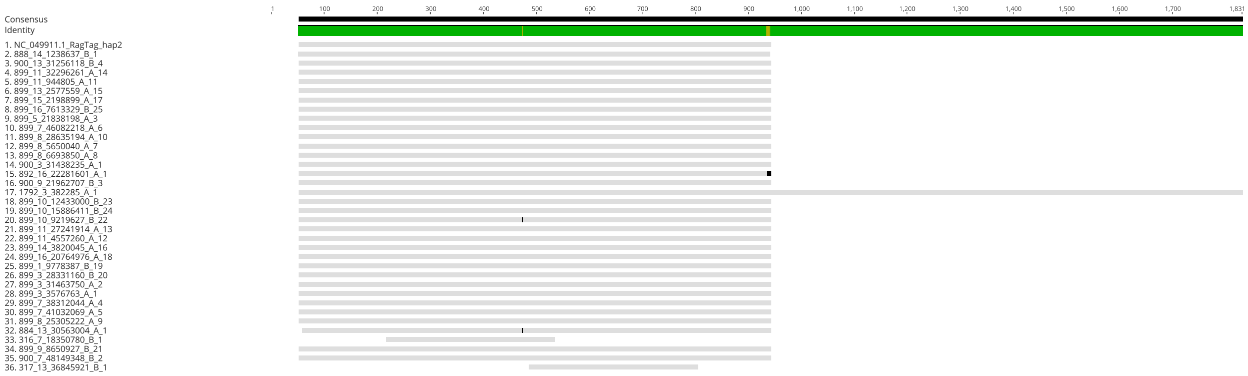

Fig. S16.
Alignment of all trimmed 900 class TEs and the matching sequence from the reference genome assembly visualized with Geneious. No open reading frames were observed, thus there is no annotation. The sequences matching consensus are represented in grey, with alternate bases displayed in black.

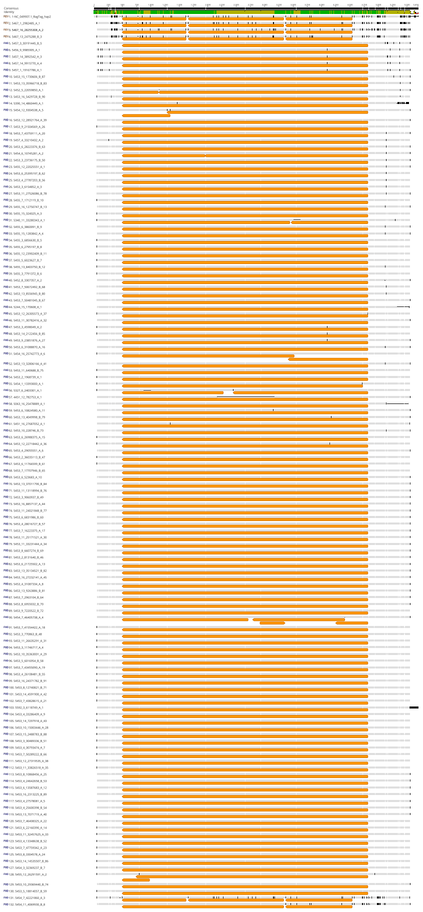

Fig. S17.
Alignment of all untrimmed 5500 class structural variants and the matching sequences from the genome assembly visualized with Geneious. Open reading frames are annotated in orange boxes underneath the sequences. The sequences matching consensus are represented in grey, with alternate bases displayed in black.

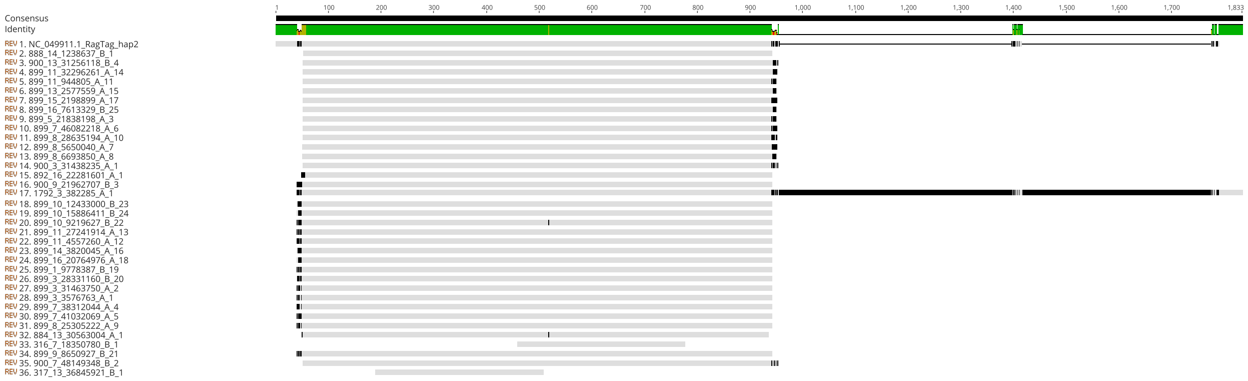

Fig. S18.
Alignment of all untrimmed 900 class structural variants and the matching sequence from the genome assembly visualized with Geneious. The sequences matching consensus are represented in grey, with alternate bases displayed in black.

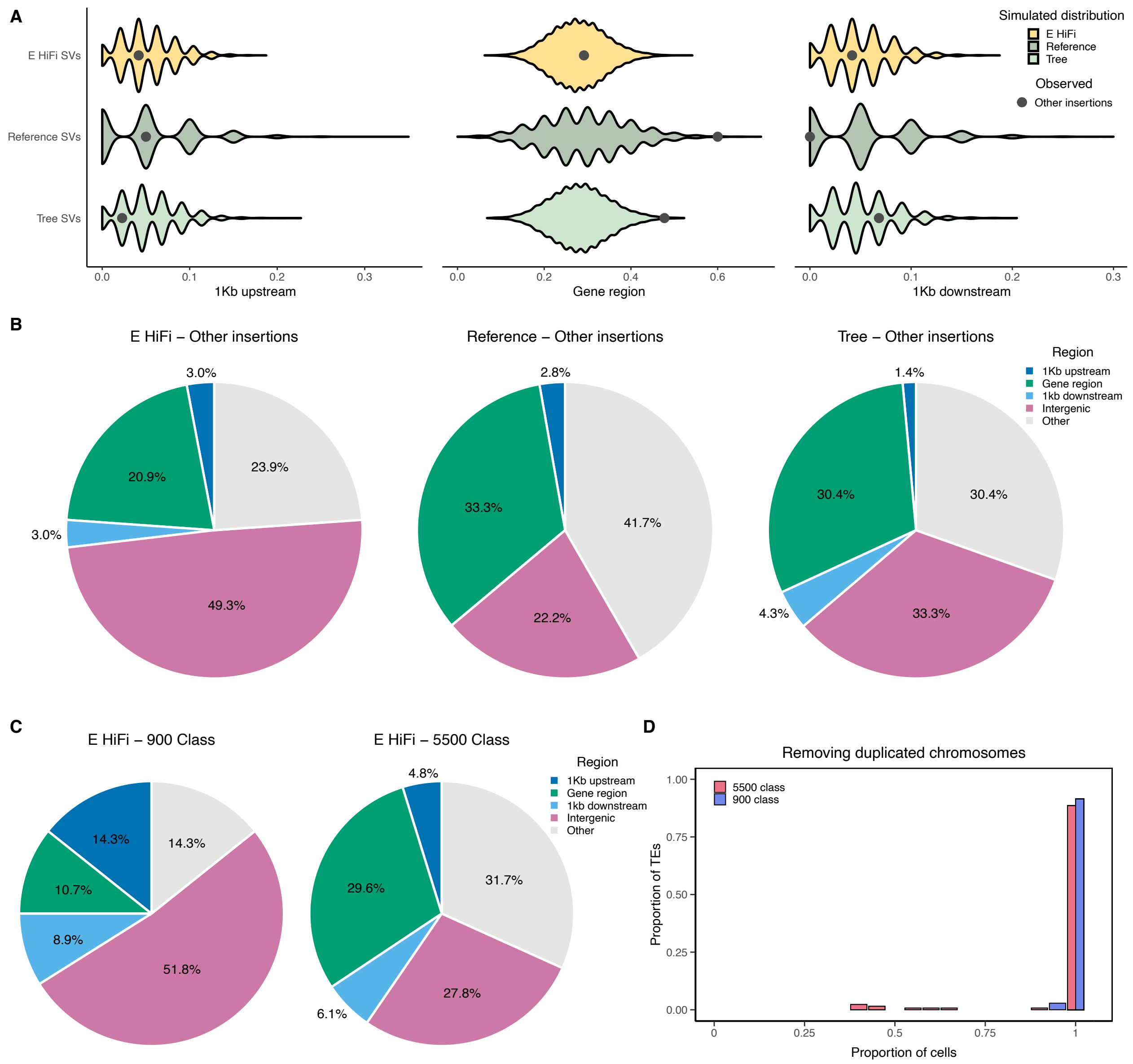

Fig. S19.
(**A**) *De novo* structural variant insertions in the long-read sequenced samples not classified as transposons were simulated to randomly insert across the genome 10,000 times, shown by the violin plots. The points indicate the observed means of the insertions. (**B**) The proportion of the *de novo* structural variant insertions not classified as transposable elements in various genomic features in the long-read sequenced samples. (**C**) The proportion of the 900 and 5500 class insertions in various genomic features in the long-read sequenced embryo. (**D**) The 900 and 5500 class TEs observed in different proportions of cells that compose the somatic embryo with the duplicated chromosomes removed.

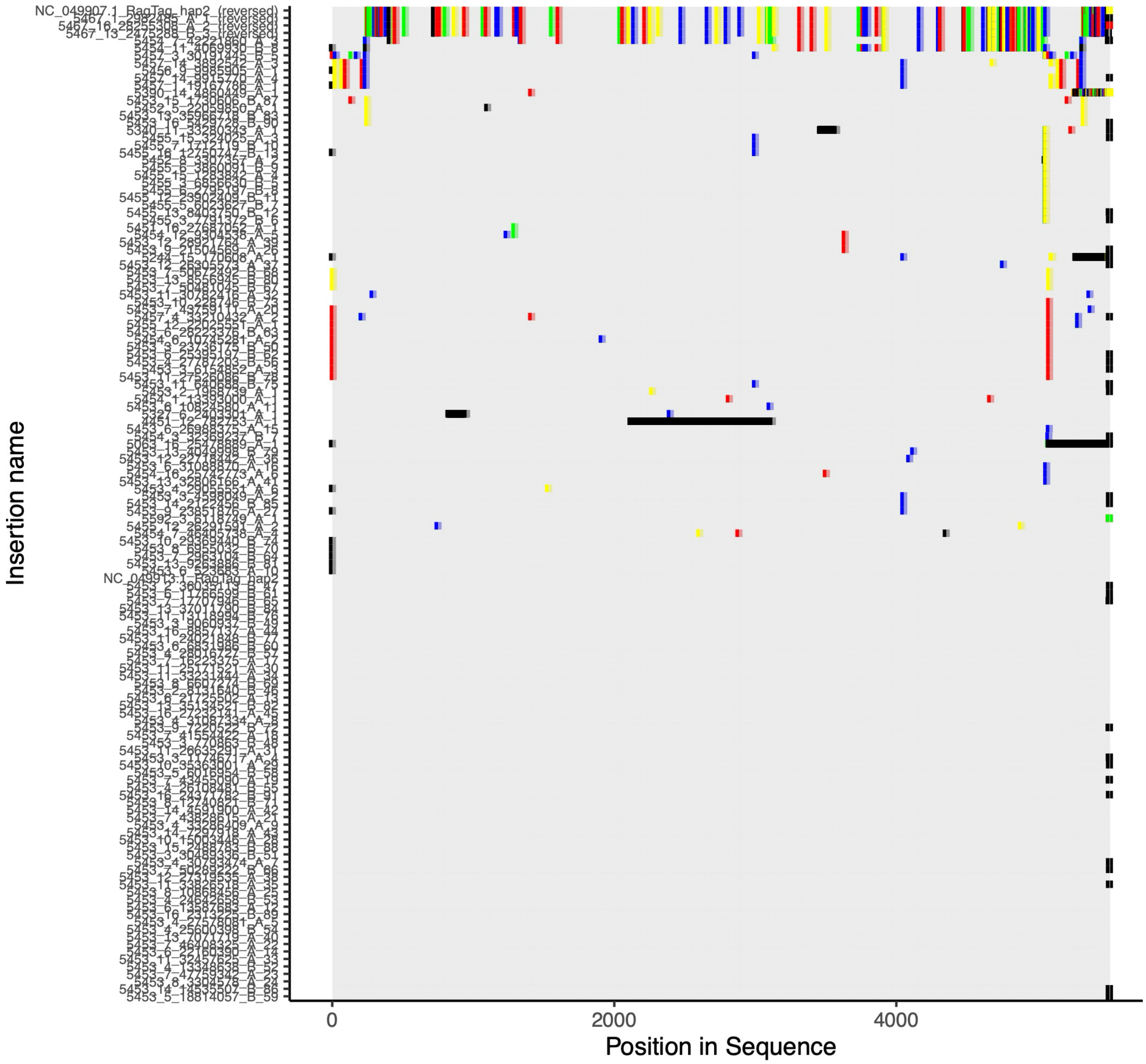

Fig. S20.
Alignment of all trimmed 5500 class TEs and the matching sequences from the genome assembly. Consensus was determined as the most common base at that position and visualized as grey. Deletions were visualized as black. Differences from the consensus of A were plotted in red, T were plotted in blue, C were plotted in green, and G were plotted in yellow. For visualization purposes, any variation from the consensus was plotted more thickly than consensus sequences.

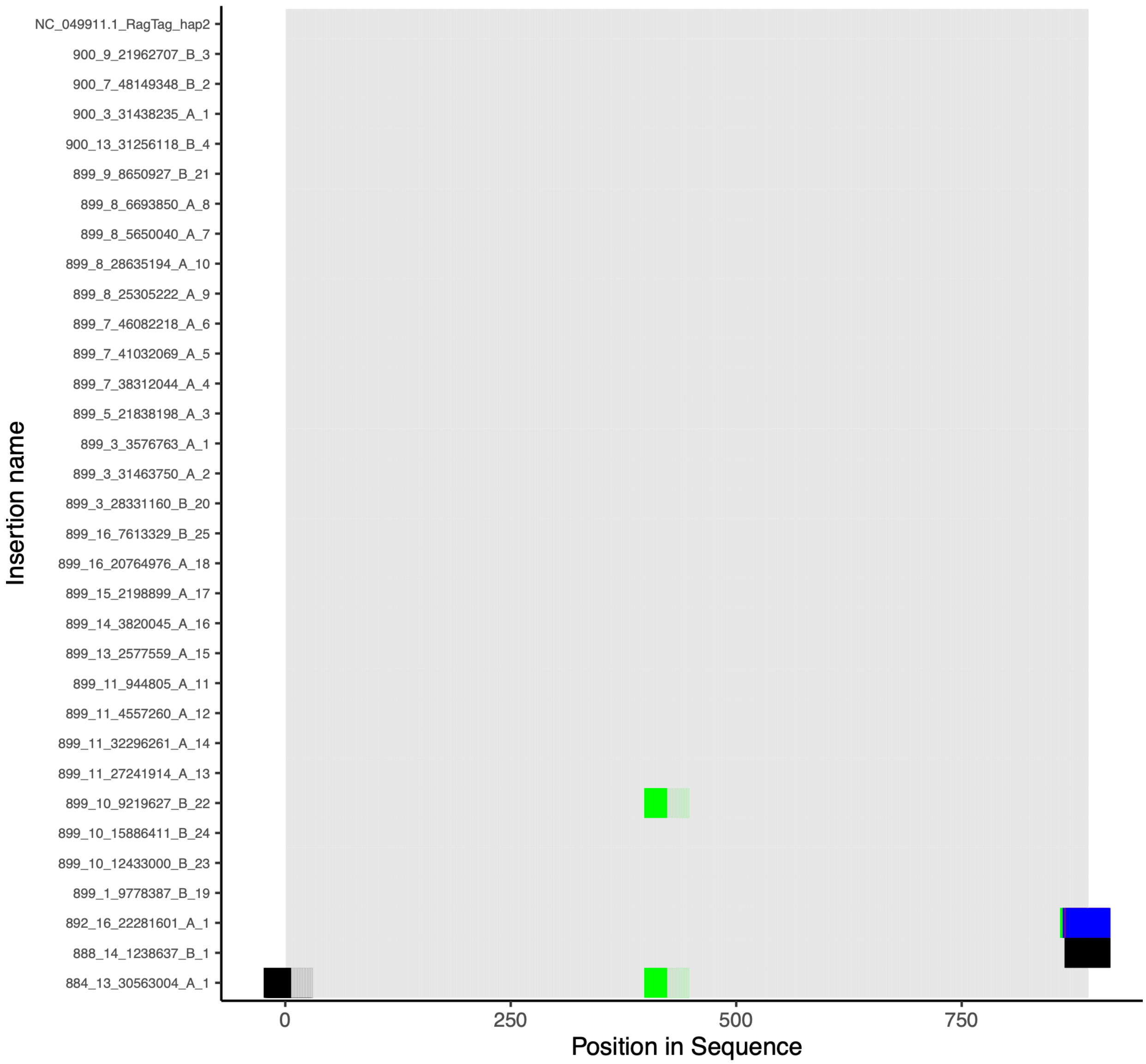

Fig. S21.
Alignment of all trimmed 900 class TEs and the matching sequence from the genome assembly. Consensus was determined as the most common base at that position and visualized as grey. Deletions were visualized as black. Differences from the consensus of A were plotted in red, T were plotted in blue, C were plotted in green, and G were plotted in yellow. For visualization purposes, any variation from the consensus was plotted more thickly than consensus sequences.

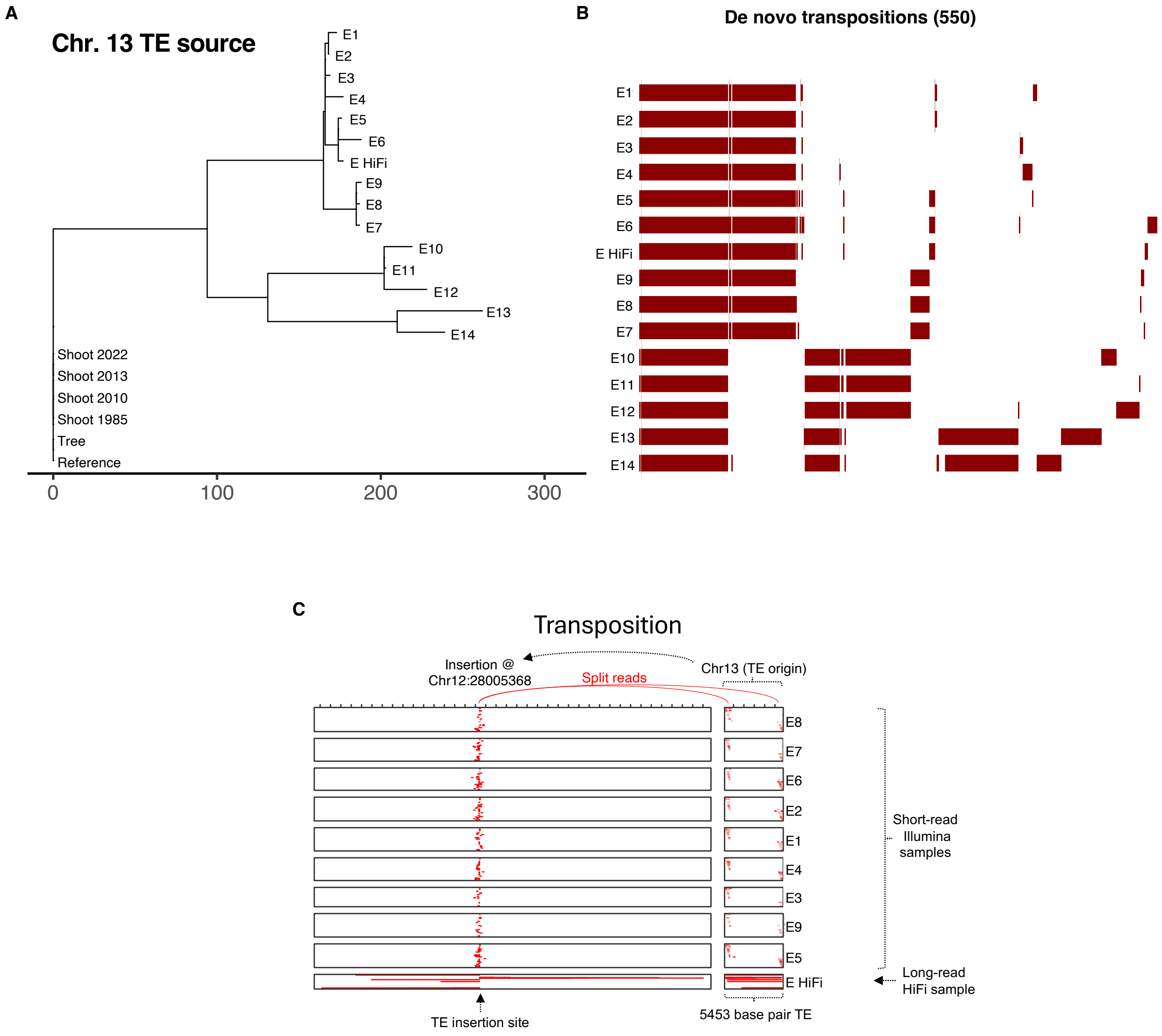

Fig. S22.
(**A**) Phylogeny of 5500 class transposable element insertion presence-absence variation in the samples. (**B**) Heatmap of 5500 class transposable element insertion presence and absence in each embryo. (**C**) Example of a detected insertion event and depiction of the detection process.

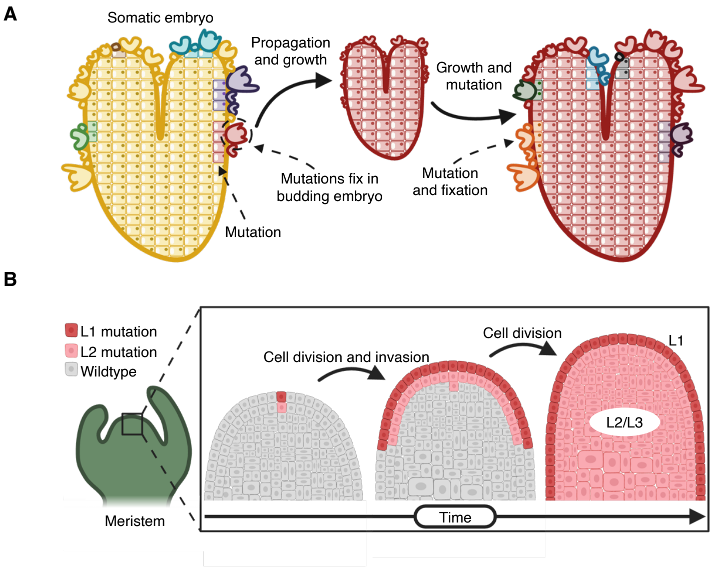

Fig. S23.
(**A**) Diagram depicting somatic embryo growth and development. A proposed mechanism of somatic mutation fixation in the somatic embryos is presented in the diagram, and somatic embryos at different developmental stages are labeled in the microscope image. (**B**) Schematic portraying a proposed mechanism of mutation fixation in the shoot culture and field-grown tree clones.

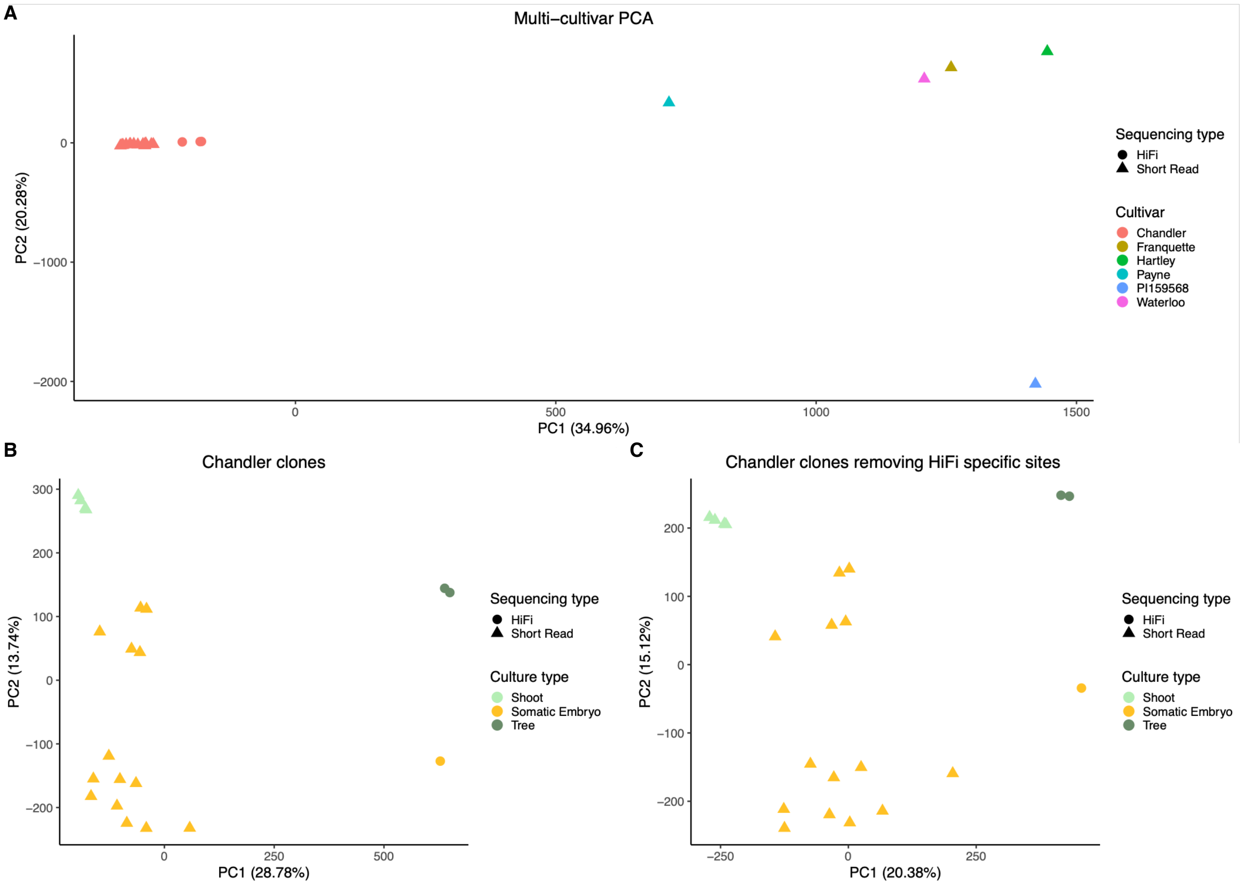

Fig. S24.
(**A**) PCA of multiple walnut cultivars and all clones. Sequencing method is denoted by shape while cultivar is denoted by color. (**B**) PCA of all clones, with shape denoting sequencing method and color denoting clonal propagation method. (**C**) PCA of all clones with sites that were called in all long-read sequenced samples and no short-read sequenced samples removed. Shape denotes sequencing method and color denotes clonal propagation method.

Supporting Information References

1. M. L. Mendum, G. McGranahan, Somatic embryogenesis of clonal "Chandler." *Walnut Res Rep, California Walnut Board* (1995).

2. G. H. McGranahan, J. A. Driver, W. Tulecke, "Tissue Culture of Juglans" in *Cell and Tissue Culture in Forestry*, (Springer Netherlands, 1987), pp. 261–271.

3. V. S. Polito, G. McGranahan, K. Pinney, C. Leslie, Origin of somatic embryos from repetitively embryogenic cultures of walnut (Juglans regia L.): Implications forAgrobacterium-mediated transformation. *Plant Cell Rep.* **8**, 219–221 (1989).

4. G. McGranahan, C. A. Leslie, J. A. Driver, In vitro propagation of mature Persian walnut cultivars. *HortScience* **23**, 220 (1988).

5. C. Leslie, G. McGranahan, "Micropropagation of Persian Walnut (Juglans regia L.)" in *Biotechnology in Agriculture and Forestry*, (Springer Berlin Heidelberg, 1992), pp. 136–150.

6. C. A. Leslie, G. H. McGranahan, Walnut micropropagation, rooting, and acclimatization. (2009).

7. S. Hu, *et al.*, Global characterization of somatic mutations and DNA methylation changes during vegetative propagation in strawberries. *Genome Res.* **34**, 1582–1594 (2024).

8. W. Xian, *et al.*, Minimizing detection bias of somatic mutations in a highly heterozygous oak genome. *G3 (Bethesda)* **15** (2025).

9. N. Wang, *et al.*, Phased genomics reveals hidden somatic mutations and provides insight into fruit development in sweet orange. *Hortic. Res.* **11**, uhad268 (2024).

10. H. Cheng, G. T. Concepcion, X. Feng, H. Zhang, H. Li, Haplotype-resolved de novo assembly using phased assembly graphs with hifiasm. *Nat. Methods* **18**, 170–175 (2021).

11. H. Cheng, *et al.*, Haplotype-resolved assembly of diploid genomes without parental data. *Nat. Biotechnol.* **40**, 1332–1335 (2022).

12. H. Cheng, M. Asri, J. Lucas, S. Koren, H. Li, Scalable telomere-to-telomere assembly for diploid and polyploid genomes with double graph. *Nat. Methods* **21**, 967–970 (2024).

13. M. Alonge, *et al.*, Automated assembly scaffolding using RagTag elevates a new tomato system for high-throughput genome editing. *Genome Biol.* **23**, 258 (2022).

14. K. Swarts, *et al.*, Novel methods to optimize genotypic imputation for low-coverage, next-generation sequence data in crop plants. *Plant Genome* **7**, lantgenome2014.05.0023 (2014).

15. J. Taylor, D. Butler, R package ASMap: Efficient genetic linkage map construction and diagnosis. *J. Stat. Softw.* **79**, 1–29 (2017).

16. M. Goel, H. Sun, W.-B. Jiao, K. Schneeberger, SyRI: finding genomic rearrangements and local sequence differences from whole-genome assemblies. *Genome Biol.* **20**, 277 (2019).

17. N. C. Durand, *et al.*, Juicer provides a one-click system for analyzing loop-resolution Hi-C experiments. *Cell Syst.* **3**, 95–98 (2016).

18. H. Li, Minimap2: pairwise alignment for nucleotide sequences. *Bioinformatics* **34**, 3094–3100 (2018).

19. H. Li, New strategies to improve minimap2 alignment accuracy. *Bioinformatics* **37**, 4572–4574 (2021).

20. M. Goel, K. Schneeberger, Plotsr: Visualizing structural similarities and rearrangements between multiple genomes. *Bioinformatics* **38**, 2922–2926 (2022).

21. F. Cabanettes, C. Klopp, D-GENIES: dot plot large genomes in an interactive, efficient and simple way. *PeerJ* **6**, e4958 (2018).

22. A. Shumate, S. L. Salzberg, Liftoff: accurate mapping of gene annotations. *Bioinformatics* **37**, 1639–1643 (2021).

23. A. Marrano, *et al.*, High-quality chromosome-scale assembly of the walnut (Juglans regia L.) reference genome. *Gigascience* **9**, giaa050 (2020).

24. S. Ou, *et al.*, Benchmarking transposable element annotation methods for creation of a streamlined, comprehensive pipeline. *Genome Biol.* **20**, 275 (2019).

25. R Core Team (2024). *R: A Language and Environment for Statistical Computing*. R Foundation for Statistical Computing, Vienna, Austria.

26. M. Manni, M. R. Berkeley, M. Seppey, F. A. Simão, E. M. Zdobnov, BUSCO update: Novel and streamlined workflows along with broader and deeper phylogenetic coverage for scoring of eukaryotic, prokaryotic, and viral genomes. *Mol. Biol. Evol.* **38**, 4647–4654 (2021).

27. G. Marcais, C. Kingsford, Jellyfish: A fast k-mer counter. *Version:* **1**, 1–4 (2012).

28. T. Ranallo-Benavidez, K. Jaron, K. Jaron, M. Schatz, M. Schatz, GenomeScope 2.0 and Smudgeplot for reference-free profiling of polyploid genomes. *Nat. Commun.* **11** (2020).

29. W. Shen, S. Le, Y. Li, F. Hu, SeqKit: A cross-platform and ultrafast toolkit for FASTA/Q file manipulation. *PLoS One* **11**, e0163962 (2016).

30. C. Pockrandt, M. Alzamel, C. S. Iliopoulos, K. Reinert, GenMap: ultra-fast computation of genome mappability. *Bioinformatics* **36**, 3687–3692 (2020).

31. N. L. Bray, H. Pimentel, P. Melsted, L. Pachter, Near-optimal probabilistic RNA-seq quantification. *Nat. Biotechnol.* **34**, 525–527 (2016).

32. Z. Sondka, *et al.*, COSMIC: a curated database of somatic variants and clinical data for cancer. *Nucleic Acids Res.* **52**, D1210–D1217 (2024).

33. L. Wang, *et al.*, The architecture of intra-organism mutation rate variation in plants. *PLoS Biol.* **17**, e3000191 (2019).

34. A. J. Orr, *et al.*, A phylogenomic approach reveals a low somatic mutation rate in a long-lived plant. *Proc. Biol. Sci.* **287**, 20192364 (2020).

35. B. T. Hofmeister, *et al.*, A genome assembly and the somatic genetic and epigenetic mutation rate in a wild long-lived perennial Populus trichocarpa. *Genome Biol.* **21**, 259 (2020).

36. S. Ossowski, *et al.*, The rate and molecular spectrum of spontaneous mutations in Arabidopsis thaliana. *Science* **327**, 92–94 (2010).

37. A. Cagan, *et al.*, Somatic mutation rates scale with lifespan across mammals. *Nature* **604**, 517–524 (2022).

38. K. A. Bird, J. C. Pires, R. VanBuren, Z. Xiong, P. P. Edger, Dosage-sensitivity shapes how genes transcriptionally respond to allopolyploidy and homoeologous exchange in resynthesized Brassica napus. *Genetics* (2023).

39. J. A. Birchler, R. A. Veitia, The gene balance hypothesis: from classical genetics to modern genomics. *Plant Cell* **19**, 395–402 (2007).

40. A. Dobin, *et al.*, STAR: ultrafast universal RNA-seq aligner. *Bioinformatics* **29**, 15–21 (2013).

41. C. Camacho, *et al.*, BLAST+: architecture and applications. *BMC Bioinformatics* **10**, 421 (2009).
